# Hypomethylation‐Triggered SERPINE1 (Serpin Family E Member 1) Exacerbates Polycystic Ovary Syndrome with Hyperandrogenism Induced by Circadian Disruption

**DOI:** 10.1002/mco2.70270

**Published:** 2025-07-04

**Authors:** Xueying Geng, Weiwei Chu, Shang Li, Xiying Zhou, Dongshuang Wang, Junyu Zhai, Yun Sun, Zi‐Jiang Chen, Yanzhi Du

**Affiliations:** ^1^ Department of Reproductive Medicine Ren Ji Hospital Shanghai Jiao Tong University School of Medicine Shanghai China; ^2^ Shanghai Key Laboratory for Assisted Reproduction and Reproductive Genetics Shanghai China; ^3^ National Research Center for Assisted Reproductive Technology and Reproductive Genetics The Key Laboratory for Reproductive Endocrinology of Ministry of Education Shandong Provincial Key Laboratory of Reproductive Medicine Center for Reproductive Medicine Shandong Provincial Hospital Shandong University Jinan China

**Keywords:** polycystic ovary syndrome, serpine1 (serpin family e member 1), DNA methylation, circadian disruption exposure, hyperandrogenism

## Abstract

Polycystic ovary syndrome (PCOS), a prevalent cause of female infertility, arises from complex interactions between genetic and environmental factors, with hyperandrogenism serving as a core pathological feature. While growing evidence links circadian disruptions to the development of hyperandrogenism in PCOS, the underlying mechanism remains unclear. In this study, we employed DNA methylation profiling and RNA sequencing of ovarian granulosa cells from rats exposed to 8‐week darkness, and identified serpin family E member 1 (SERPINE1) as a key player. SERPINE1 was significantly hypomethylated and upregulated in the dark group, correlating with elevated androgen levels. Mechanistically, using CRISPR–dCas9‐based targeted methylation, we found that CpG hypomethylation near the SERPINE1 transcription start site drove its overexpression. Functional assays revealed that SERPINE1 suppression activated the PI3K/AKT signaling pathway, thereby enhancing CYP19A1 expression and enzymatic activity to facilitate androgen conversion in vitro. Moreover, treatment with the SERPINE1 inhibitor tiplaxtinin alleviated both reproductive and metabolic abnormalities in rat models treated with either dehydroepiandrosterone or exposed to darkness. These findings highlight SERPINE1's role in circadian disruption‐induced hyperandrogenism and its potential as a methylome‐based diagnostic biomarker for PCOS. Pharmacological inhibition of SERPINE1 emerges as a promising therapeutic strategy for hyperandrogenic PCOS.

## Graphical abstract

SERPINE1 shows CpG hypomethylation near its TSS of promoter region when exposed to circadian disruption, and its gene expression triggers hyperandrogenism through the PI3K/AKT pathway. SERPINE1 inhibitor TPX can significantly decrease serum SERPINE1 levels and reduce abnormally high testosterone levels in both continuous darkness‐exposed rats and DHEA‐treated rats.

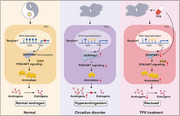

## 1 Introduction

Polycystic ovary syndrome (PCOS) is the leading cause of female infertility and affects approximately 6–20% of women of reproductive age worldwide [1, 2]. It has highly heterogeneous clinical symptoms. Many women with PCOS suffer from hyperandrogenism, chronic anovulation, and severe health consequences, such as reduced fertility and type 2 diabetes mellitus [3]. Hyperandrogenism, the hallmark feature of PCOS, plays a pivotal role in the disease development [4], affecting the patient's reproductive and metabolic function and aggravating pregnancy complications [5]. Precise PCOS management is hampered by the clinical heterogeneity of the disease as well as the lack of a clear mechanistic etiology and specific prognostic markers. Therefore, other factors involved in the pathogenesis of hyperandrogenism in PCOS need to be determined to identify unique pharmacological targets.

PCOS is caused by a combination of genetic and environmental factors. There is growing evidence that environmental exposure and lifestyle issues, particularly circadian disruption, are strongly associated with PCOS development [6, 7, 8]. Many women with PCOS experience sleep disturbances and circadian disruption [9, 10], abnormal circadian gene expression in peripheral blood mononuclear cells and ovarian granulosa cells (GCs), and abnormal androgen metabolism gene expression [6, 7]. However, the causal relationship between hyperandrogenism and circadian disruption in PCOS as well as the underlying mechanisms has not been clearly established. Our previous studies developed a continuous darkness‐exposed rat model using the nocturnal nature of rats to simulate prolonged nocturnal activity in humans. This model recapitulates most of the diagnostic criteria for PCOS, including hyperandrogenism, estrous cycle dysfunction, polycystic ovary, and insulin resistance [11, 12]. We expect this model to facilitate the identification of mechanisms underlying the etiology of PCOS.

Epigenetic modifications, such as DNA methylation, bridge the gap between nature and nurture as they can be shaped by environmental exposures that increase disease susceptibility. Women with PCOS exhibit abnormal DNA methylation [13]. Interestingly, the methylation of genes associated with reproductive function, ovarian steroidogenesis, glucose and lipid metabolism, adipose tissue activity, inflammation, and immune response regulation has been suggested to be involved in PCOS development [14, 15]. However, little is known about the specific DNA methylation targets and pathways involved in circadian disruption and PCOS development.

In this study, we aimed to determine whether and how circadian disruption affects the dynamics of the DNA methylome and transcriptome in ovarian GCs, potentially leading to PCOS‐like phenotypes in rats. We performed target‐captured methylation sequencing [16] and RNA‐seq analysis in rats with 8‐week continuous darkness exposure and controls. Our goal was to identify the methylation and transcriptional molecular networks involved in circadian disruption and their role in regulating PCOS development. Next, we focused on serpin family E member 1 (SERPINE1), a key gene hypermethylated and highly expressed in the model. SERPINE1, also known as plasminogen activator inhibitor‐1 (PAI‐1), is a glycoprotein that modulates extracellular matrix remodeling and fibrinolysis, which was later found to be correlated with various reproductive diseases [17]. In the ovary, SERPINE1 is predominantly localized in GCs and theca cells. Overexpression of human SERPINE1 in mice has been shown to induce cystic follicle formation and elevate serum testosterone levels [18]. Clinical studies in PCOS patients have further demonstrated that plasma SERPINE1 levels are elevated in both normal‐weight and overweight/obese women with PCOS compared with BMI‐matched controls [19]. In vitro experiments have revealed that SERPINE1 can impair glucose metabolism in ovarian GCs [20]. These findings collectively suggest that SERPINE1 plays a significant role in androgen synthesis and metabolism.

In our study, we confirmed that SERPINE1 expression was significantly upregulated in both circadian disruption model animals and PCOS patients, and its levels were positively correlated with testosterone concentrations. We observed that SERPINE1 was epigenetically regulated by DNA hypomethylation in the promoter region in an anomalous circadian environment. In turn, high SERPINE1 expression led to androgen accumulation through the PI3K/AKT pathway by reducing aromatase expression in GCs. We provide evidence that SERPINE1 inhibitors used to treat rats with circadian disruption and PCOS rescued the reproductive endocrine and metabolic alterations of PCOS. This study revealed a promising epigenetic diagnostic biomarker as well as a therapeutic target for PCOS.

## 2 Results

### 2.1 The Continuous Darkness‐Exposed Rat Model Evolved PCOS‐Like Phenotypes

Our previously studies revealed that 8‐week darkness exposure to rats resulted in PCOS‐like phenotypes, including hyperandrogenism, disturbances in the estrous cycle, and polycystic ovarian [11, 12], which prompted us to investigate when individual phenotypes emerged and how they dynamically changed.

**FIGURE 1:**
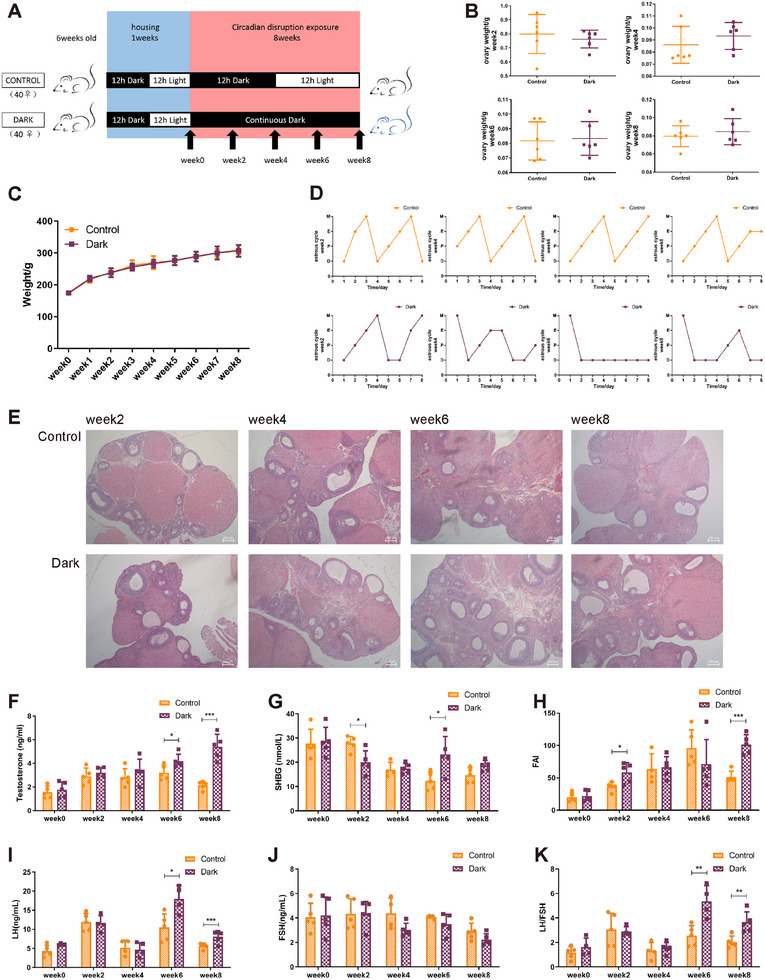
Continuous dark exposure induces multiple reproductive endocrine traits of PCOS. (A) Schematic illustration of the modeling design. (B) Ovarian mass in continuous dark rats versus controls (*n* = 6). (C) Body mass of dark rats and controls that measured weekly over 8 weeks (week0: *n* = 40, week1 and week2: *n* = 32, week3 and week4: *n* = 24, week5 and week6: *n* = 16, week7 and week8: *n* = 8). (D) Representative estrous cycles of 8 rat/group during 8 consecutive days using a vaginal smear. M: metestrus, E: estrus, P: proestrus, D: diestrus phase. (E) Representative images of ovaries stained with H&E from dark rats versus controls (*n* = 3), scale bars, 200 µm. (F–K) Levels of testosterone (F), SHBG (G), LH (I), FSH (G) in rat serum are measured using ELISA. FAI (H) is calculated as FAI = T/SHBG × 100%. LH/FSH ratio (K) is calculated as LH divided by FSH (*n* = 4–5). SHBG, sex hormone binding globulin; LH, luteinizing hormone; FSH, follicle stimulating hormone; FAI, free androgen index. In (B)–(C) and (F)–(K), data are represented as mean ± SD. *p < 0.05, **p < 0.01, ***p < 0.001 (Student's *t* test).

To elucidate the longitudinal alterations in the continuous darkness model, 6‐week‐old female SD rats were randomly divided into two groups after 1 week of acclimatization: control group, exposed to normal 12:12 h light–dark cycle, and dark group, exposed to continuous darkness. At weeks 0, 2, 4, 6, and 8, the rats were sacrificed for observation and sampling (Figure 1A). No changes were observed in their ovarian mass or body weight during the 8‐week period (Figure 1B,C). Next, the appearance of reproductive endocrine alterations was evaluated. As expected, the dark group exhibited major phenotypes in the PCOS diagnostic criteria, including irregular estrous cycles (Figure 1D), polycystic ovary (Figure 1E), and hyperandrogenism (Figure 1F). Notably, the testosterone levels of the dark group increased in a time‐dependent manner, with a significant difference at week 6 and a further increase at week 8 (Figure 1F). Although there was no significant change in sex hormone‐binding globulin (SHBG) levels at any point within 8 weeks (Figure 1G), it was postulated that the free androgen index (FAI) was higher at weeks 2 and 8 in the dark than in the control group (Figure 1H). Furthermore, luteinizing hormone (LH) levels significantly increased at weeks 6 and 8 whereas the follicle‐stimulating hormone (FSH) levels remained unchanged and the LH/FSH ratio significantly increased (Figure 1I–K). The dark group also exhibited impaired inflammation and lipid metabolism at week 8 (Figure S1). These findings indicate that phenotypes in rats with prolonged darkness exposure emerge at different time points, implying the existence of various epigenetic regulatory mechanisms governing acute response and chronic adaptation.

### 2.2 Continuous Darkness Exposure Leads to Dynamic Methylomic and Transcriptomic Profiles Changes in Ovarian GCs

**FIGURE 2:**
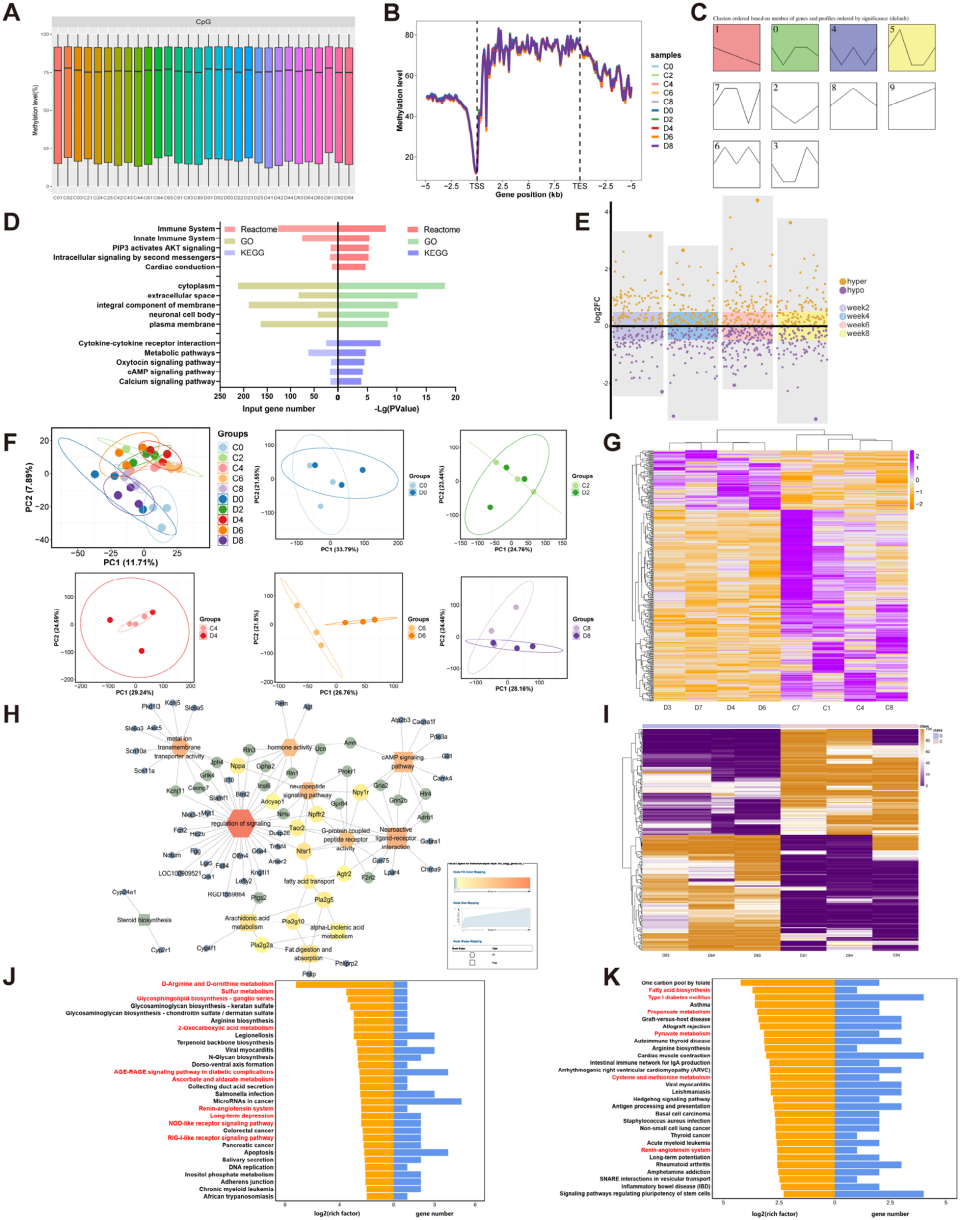
Integrated analyses of the DNA methylome and RNA sequencing of rat granulosa cells. (A) Boxplot showing the overall distribution of the methylation of all CpG loci in each rat GCs sample. The short bold bar in the box represents average methylation. Sample numbers are stated in the bottom of each box. *p* Values are calculated using a two‐tailed Student's *t* test. (B) Average distribution of DNA methylation level for each group of samples plotted on a gene model. TSS, transcription start site; TES, transcription end site. C0, control group in week 0; D0, dark group in week 0, and so on. (C) Short time‐series expression miner (STEM) analysis in dark group over 8 weeks. Four clusters were identified significantly and the average promoter methylation patterns were visualized. Line plots are used to show fold changes (log2FC). (D) Functional annotation charts using Reactome/GO/KEGG performed on the 1608 genes in first cluster (red) of STEM analysis (C). Significance is indicated as −log10 *p* value. (E) DMR genes from week 2 to week 8. Orange is hypermethylation and purple is hypomethylation in the dark group compared with the control group. The spots are shown as fold changes (log2FC). DMR, differentially methylated regions. (F) PCA of the variance in methylation levels of CpG sites. C0, control group in week 0; D0, dark group in week0, and so on. PCA, principal components analysis. (G) Heatmap for RNA sequencing of GCs from dark group and controls at week 6 (|Log2FC|≥1.5, *p* < 0.05). (H) Network visualization of enriched GO terms and KEGG pathways for DEGs at week 6. Hexagons/squares represent terms/pathways, with nodes (circles) representing genes. Similar gene sets are connected by lines (edges), with distance indicating inverse overlap. Functional clustering (color or size) is based on kappa statistics. DEG, differentially expressed genes. (I) Heatmap for DNA methylation target‐captured bisulfite sequencing of ovarian granulosa cells (GCs) from dark group and controls at week 6 (|Log2FC|>0.5, *p* < 0.05). (J–K) Functional enrichment charts using KEGG on the genes associated with hypomethylated sites (J) and hypermethylated sites (K) at week 6. Top 30 highest rich factor pathways are shown.

To identify the epigenetic regulatory mechanism responsible for the dynamic phenotypes at different time points of continuous darkness, SureSelectXT rat DNA methylation target‐captured bisulfite sequencing (MC‐seq; Agilent Technologies, CA, USA) was employed, and the DNA methylation of rat ovarian GCs collected at five time points spanning 8 weeks was examined (Figure 1A). Genome‐wide DNA methylation profiles indicated that 15–85% of tested CpG sites were methylated (binomial test, *q*‐value < 0.05), consistent with previous findings [21]. Global DNA methylation showed only mild overall changes over time (Figure 2A). Subsequently, each set of standardized DNA methylation data was aligned to a gene model that includes promoting/transcribing starting points (TSSs) and transcribing ending points. The methylation patterns of the 10 groups were homologous. And there was a sharp dip in methylation levels 1 kb upstream of the TSS, followed by a nadir upstream of the TSS and high methylation levels throughout the gene body region (Figures 2B and S2A,B). This indicates that DNA methylation profiles were generally longitudinally stable and that 8‐week continuous darkness exposure would not cause significant disturbance to DNA methylation.

To identify the dynamic patterns of longitudinal methylation profiles, we focused on scrutinizing the methylation levels in gene promoter regions via short time‐series expression miner (STEM) analysis. The results indicated four clustering modules in the dark group (*p* < 0.05) (Figure 2C). The first module was progressively hypomethylated over time, which exhibited a similar methylation profile to patients with PCOS [13] and people working in night shift [22]. Subsequently, we performed Gene Ontology (GO) enrichment, Kyoto Encyclopedia of Genes and Genomes (KEGG), and Reactome Pathway analyses to predict the function and pathway profile of this module with hypomethylation trends. The genes were mainly enriched in immune inflammation and the AKT and metabolic pathways (Figure 2D). The STRING protein interaction network (http://string‐db.org/) demonstrated that persistently hypomethylated genes were mostly enriched in the immune system, encompassing antigen presentation, ubiquitination modifications, and inflammatory factors (Figure S2C–G). To investigate the association between differentially methylated sites (DMSs) and each phenotype, weighted correlation network analysis was conducted to obtain three modules (MEdarkorange, MEgrey60, and MEblack) exhibiting significant and negative associations with testosterone, FAI, LH, leptin, and interleukin 1 beta (IL‐1b). Furthermore, KEGG analysis revealed differentially expressed genes (DEGs) within the modules enriched in metabolism‐related pathways (Figure S2H–J). These data suggest that changes caused by darkness are enriched in neural, metabolic, and inflammatory pathways.

Although the average differences of methylation sites across all time points were small, 386–511 differentially methylated regions (DMRs) and 80,926–86,927 DMSs were identified between each dark and control group pair (Figure S3A,B). These may hold the key to driving disease phenotypes. Dynamic volcano plots revealed a significant increase in DMSs at week 6 and a significant elevation in hypomethylated sites in the dark group (Figure 2E). Notably, the highest number of DMRs and DMSs in promoter TSS regions was still observed in week 6 (Figure S3C,D). We herein demonstrate the changes in the CpG sites in the TSS region of the genes Igf2r and timeless over the time course. Although the methylation changes varied from gene to gene, their differences expanded at week 6 (Figure S3E,F). Principal component analysis revealed similar phenomenon that rats at week 6 were better clustered according to the CpG site methylation (Figure 2F). As the most notable methylation variations occur during week 6, it will be of great interest to investigate the progressive alteration‐induced expression changes at this point.

We remodeled rats in continuous darkness for 6 weeks to extract RNA from ovarian GCs for RNA sequencing. Compared with the control group, the dark group had similar body weights (Figure S4A), notable abnormalities in testosterone levels (Figure S4B), ovarian histological sections (Figure S4C), and glucose metabolism (Figure S4D,E). A total of 378 DEGs were identified between the dark and control groups, including 90 and 288 up‐ and downregulated genes, respectively (Figure 2G). Functional enrichment and clustering analyses of the DEGs revealed enrichment of terms related to lipid metabolism and neural pathways (Figure 2H). This finding is consistent with the results of the enrichment analysis of DNA methylation changes induced by 6‐week darkness exposure (Figure 2I–K).

### 2.3 Darkness‐Induced Serpine1 CpGs Hypomethylation Near TSS is Highly Correlated with Serum Testosterone Levels

**FIGURE 3:**
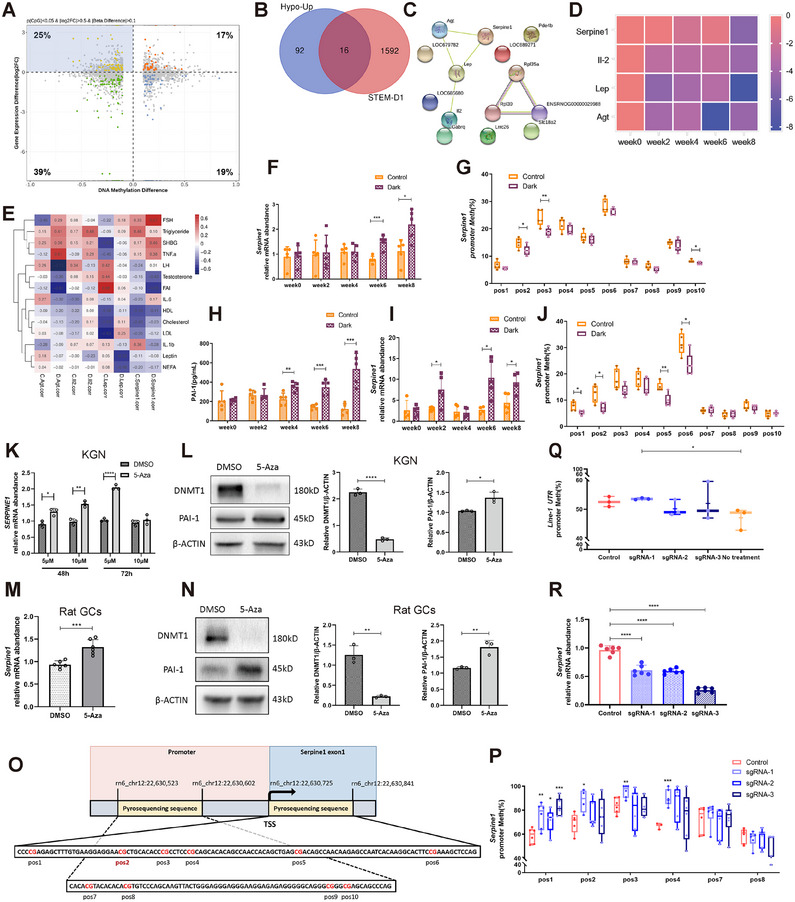
Decreased DNA methylation with concomitant increased gene expression of Serpine1 was highly associated with serum testosterone. (A) Scatterplot of promoter differentially methylated site (DMS) genes versus DEGs of dark group and controls at week 6. Significant correlation genes are marked in colors (red, yellow, green and blue), respectively (p(CpG) < 0.05, p(DEG) < 0.05, log2FC > 0.5, beta difference > 0.1. Fisher's exact test, *p* < 0.05). (B) Venn diagram of the 108 genes with promoter hypomethylation and upregulated expression in Quadrant II (A, Hypo‐Up) and the 1608 genes in first cluster (red) of STEM analysis (Figure 2C, STEM‐D1), with 16 genes overlapping. (C) STRING protein network analysis of the 16 common genes from the Venn diagram (B). (D) Heatmap for average gene promoter methylation signatures of dark groups over 8 weeks. The four candidate genes were obtained from Figure 3C. (E) Correlation analysis between average promoter methylations and phenotypes for the 4 genes at week 6. (F) qPCR analysis for Serpine1 expression in GCs of dark and control rats over 8 weeks (*n* = 5). (G) Pyrosequencing to detect methylation status of Serpine1 promoter CpG sites in the GCs at week 6 (*n* = 5). Pos, CpG site in Serpine1 promoter; Meth, methylation level. The positions of the ten CpG sites are shown in (O). (H) ELISA assay to detect changes in serum PAI‐1 levels in dark group and controls during 8 weeks (*n* = 5). (I) qPCR analysis for expressions of Serpine1 in rat liver during 8 weeks (*n* = 4–5). (J) Pyrosequencing to detect methylation status of Serpine1 promoter CpG sites in rat liver at week 6 (*n* = 5). The positions of the ten CpG sites are shown in (O). (K–N) qPCR analysis of SERPINE1 expression in KGN cells (K, *n* = 3) or rat primary GCs (M, *n* = 6) treated with 5‐Aza (5‐Aza‐2′‐deoxycytidine, 5 µM, 10 µM) for 48 or 72 h, respectively. Western blot analysis of PAI‐1 and DNMT1 expression normalized to β‐ACTIN protein levels in KGN cells (L) or rat primary GCs (N) treated with 5‐Aza (5 µM) for 72 h, respectively (*n* = 3). The quantification of protein expression is presented on the right. (O) Schematic illustration of pyrosequencing sequences. Pos2 is a DMS loci. (P) Pyrosequencing to detect methylation status of Serpine1 promoter CpG sites in BRL‐3A cells transduced with three different dCas9–Dnmt3aCD/sgRNAs or dCas9–Dnmt3aCD/control (*n* = 4–6). The positions of the ten CpG sites are shown in (O). (Q) Pyrosequencing to detect methylation status of the 5' untranslated region (UTR) of LINE‐1 elements in BRL‐3A cells transduced with three different dCas9–Dnmt3aCD/sgRNAs, dCas9–Dnmt3aCD/control, or no treatment (*n* = 3). (R) qPCR analysis of Serpine1 expression in BRL‐3A cells of the four groups (*n* = 6). In (F)–(N) and (P)–(R), data are represented as mean ± SD. *p < 0.05, **p < 0.01, ***p < 0.001, ****p < 0.0001. (F)–(N): Student's *t* test; (P)–(R): one‐way ANOVA.

To further explore the association between this epigenetic modification and expression changes induced by 6‐week darkness exposure, we conducted an integrative analysis between 2818 DEGs and 4272 promoter DMSs. Notably, of the four quadrants, quadrant II showed 108 genes with reduced promoter CpG methylation and higher expression (Figure 3A). Venn analysis was conducted between genes in quadrant II and those with persistent hypomethylation in STEM‐D1, identifying 16 overlapping genes. These genes maintained sustained hypomethylation levels while being overexpressed during the 6‐week darkness exposure (Figure 3B). Notably, four candidate genes, *Serpine1* [17], *Lep* [23], *IL‐2* [24], and *Agt* [25], encoding secretory proteins and having evident associations with inflammation and lipid metabolism were clustered in the STRING network (Figure 3C). The heat map showed a gradual trend of decreasing methylation in the promoter region of these four genes over time (Figure 3D). Interestingly, significantly negative correlations were observed between the promoter region methylation of *Serpine1* and both serum testosterone and FAI (Figure 3E), suggesting a potential association between *Serpine1* methylation and androgens. These findings suggest that hypomethylation contributes to *Serpine1* overexpression and, hence, hyperandrogenism.

Several studies have reported inverse associations between DNA methylation and gene transcription levels in *SERPINE1* [26]. However, research on specific *Serpine1* CpGs that can induce transcriptional changes is lacking. We validated *Serpine1* overexpression over 8 weeks in the GCs via qPCR (Figure 3F). Furthermore, we confirmed hypomethylation of the CpG (position 2) observed in MC‐seq and nearby CpGs from the *Serpine1* promoter region via pyrosequencing (Figure 3G,O), followed by a gradual increase in serum PAI‐1 levels via ELISA (Figure 3H). This finding was also confirmed in the liver (Figure 3I,J), the main organ involved in *Serpine1* synthesis [27], as well as in adipose tissues (Figure S5A) and brown adipose tissues (Figure S5B). These data indicate that hypomethylation and high *Serpine1* expression are universal darkness‐induced phenomena, suggesting that DNA methylation plays a pivotal role in the regulation of *Serpine1* expression.

To further validate this methylation modification switch‐based gene activation, we treated human KGN cell line cells with 5‐Aza‐2′‐deoxycytidine (5‐Aza), a DNA methyltransferase inhibitor that binds to DNA and inhibits DNMT activity and expression [27]. qPCR and Western blotting revealed that 5‐Aza promoted SERPINE1 expression in a dose‐ and time‐dependent manner (**Figure** **3K**,**L**). Similar results were observed in primary rat GCs (Figures 3M,N and S5C).

Next, we used a targeted DNA methylation editing tool, dCas9–Dnmt3aCD, for epigenetic alterations at specific CpG sites to infer a cause–effect relationship. Specific guide RNAs (sgRNA) were designed to bring the dCas9–DNMT3aCD system to the sequence located near the TSS of the first exon of *Serpine1*, including positions 1, 2, and 3 in the rat normal liver cell line BRL‐3A (Figures 3O and S5D). Pyrosequencing revealed that dCas9–Dnmt3aCD/gRNA transduction significantly increased DNA methylation at positions 1, 2, and 3 and at adjacent position 4 but not at distant positions 7 and 8 (Figure 3P). DNA methylation of long interspersed nuclear element 1, which reflects global DNA methylation in cells, did not significantly differ among the sgRNA treatments and controls (Figure 3Q). Chromatin‐immunoprecipitation results also validated the specific binding of the DNMT3A protein to the target promoter region (Figure S5E). These results indicated successful on‐target methylation. Subsequently, qPCR results showed a significant reduction in *Serpine1* transcripts in targeted methylated cells (Figure 3R). These results suggest that *Serpine1* expression was efficiently decreased by methylation near the TSS.

### 2.4 SERPINE1 Inhibits Aromatase Expression and Promotes Androgen Accumulation Through the PI3K/AKT Pathway

**FIGURE 4:**
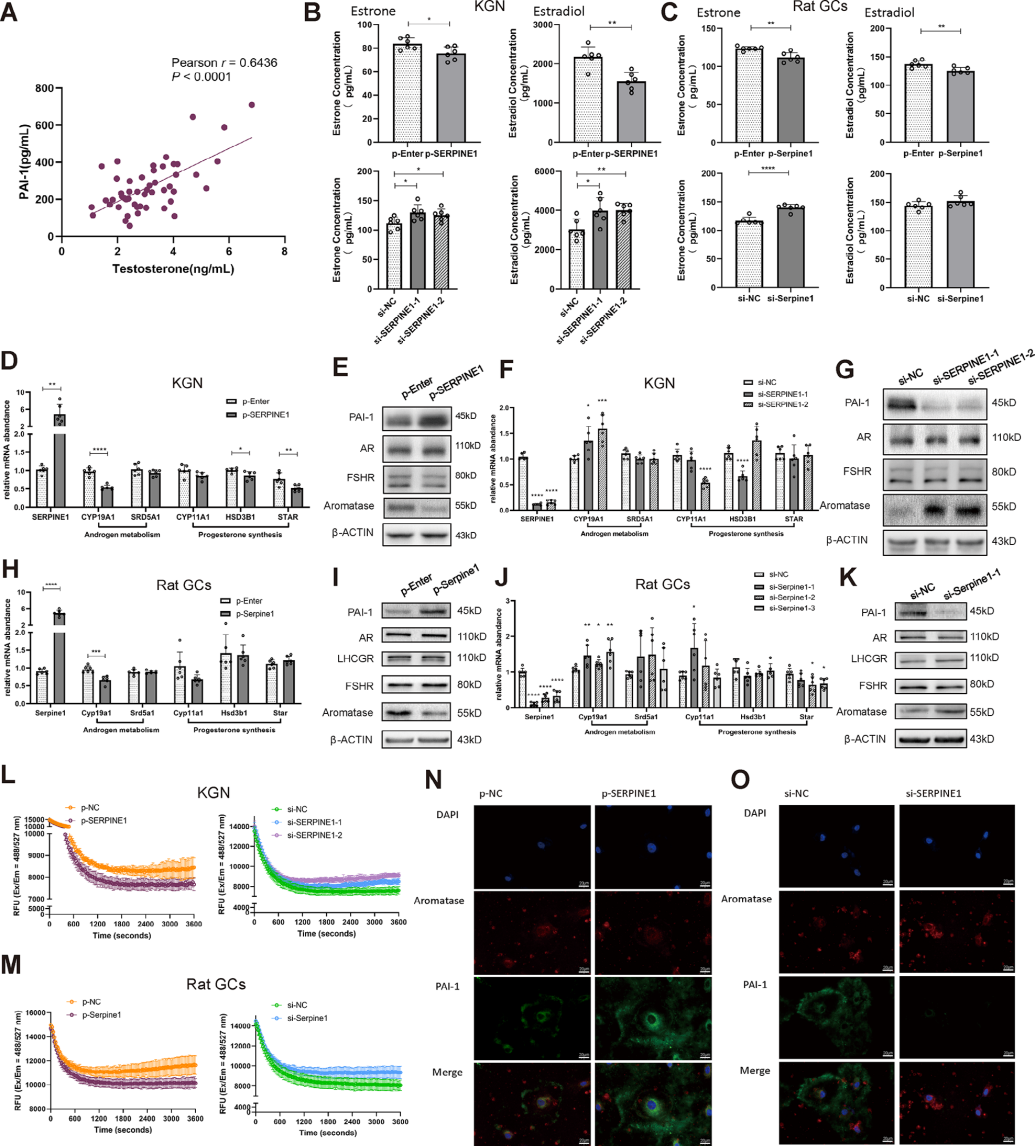
Serpine1 represses aromatase expression and activity. (A) Pearson analysis between serum testosterone and PAI‐1 levels from rats in all 8 weeks (*n* = 50, Pearson *r* = 0.6436, *p* < 0.0001). (B and C) Estradiol or estrone levels in the cell supernatant of KGN cells (B) or rat GCs (C) were measured by ELISA after overexpression of SERPINE1 (upper 2 panels) or knockdown of SERPINE1 (lower 2 panels) followed by addition of human menopausal gonadotropin (hMG, 500 mIU/mL) and testosterone (10 µM) or androstenedione (10 µM) for 4 h (*n* = 6). (D, H, F, and J) qPCR analysis of expression of androgen‐metabolism‐related genes and progesterone‐synthesis‐related genes after SERPINE1 overexpression in KGN cells (D) or rat GCs (H) or after SERPINE1 knockdown in KGN cells (F) or rat GCs (J) (*n* = 6). (E, I, G, and K) Western blot analysis of protein levels of PAI‐1, aromatase, and other androgen‐metabolism‐related markers (LHCGR is rarely expressed in KGN cells) normalized to β‐ACTIN protein levels after SERPINE1 overexpression in KGN cells (E) or rat GCs (I) (E, *n* = 6, I, *n* = 3) or after SERPINE1 knockdown in KGN cells (G) or rat GCs (K) (*n* = 3). (L and M) The aromatase activity of KGN cells (L) or rat primary GCs (M) detected by aromatase (CYP19A) activity assays after SERPINE1 overexpression (left) or SERPINE1 knockdown (right) followed by addition of hMG (500 mIU/mL) and testosterone (10 µM) or androstenedione (10 µM) for 4 h (*n* = 3). (N and O) Representative images of Aromatase expression (red) and PAI‐1 expression (green) in SERPINE1‐overexpressing (N) or ‐knocked‐down (O) KGN cells using immunofluorescence staining. Images are representative of three independent experiments. Nuclei were counterstained with DAPI. Scale bars, 20 µm. In (B)–(D), (F), (H), and (J), data are represented as mean ± SD. *p < 0.05, **p < 0.01, ***p < 0.001, ****p < 0.0001. (A): Pearson *r* test, (B)–(D), and (H): Student's *t* test; (F) and (J): one‐way ANOVA.

Since multiomics sequencing results indicated a potential correlation between *Serpine1* and testosterone, we conducted Pearson's analysis on serum SERPINE1 (PAI‐1) and testosterone and found a significantly positive correlation (Figure 4A).

Therefore, investigating the function of SERPINE1 in the androgen metabolic pathway in GCs is valuable. First, we conducted an indirect assay for aromatase activity by detecting estrogen levels in cell supernatants after adding androgen, and found a significant decrease in aromatase activity after SERPINE1 overexpression and vice versa after knockdown in either KGN or primary rat GCs (Figure 4B,C). Second, we examined the expression of several key enzymes involved in the androgen metabolic pathway. CYP19A1 expression significantly decreased after SERPINE1 overexpression and vice versa after knockdown (Figures 4D–K and S6). Third, we used an activity assay kit to directly detect aromatase activity and the results were consistent with those with the indirect method (Figure 4L,M). Using immunofluorescence, we verified that SERPINE1 inhibited CYP19A1 expression, and identified that SERPINE1 was mainly localized in the cytoplasm and extracellular matrix; instead, aromatase was distributed in the nucleus and cytoplasm, with more in the cytoplasm (Figure 4N,O). Accordingly, we hypothesized that SERPINE1 in the cytoplasm and extracellular matrix might transmit repressive signals through specific signaling pathways, thereby inhibiting CYP19A1 transcription and expression.

**FIGURE 5:**
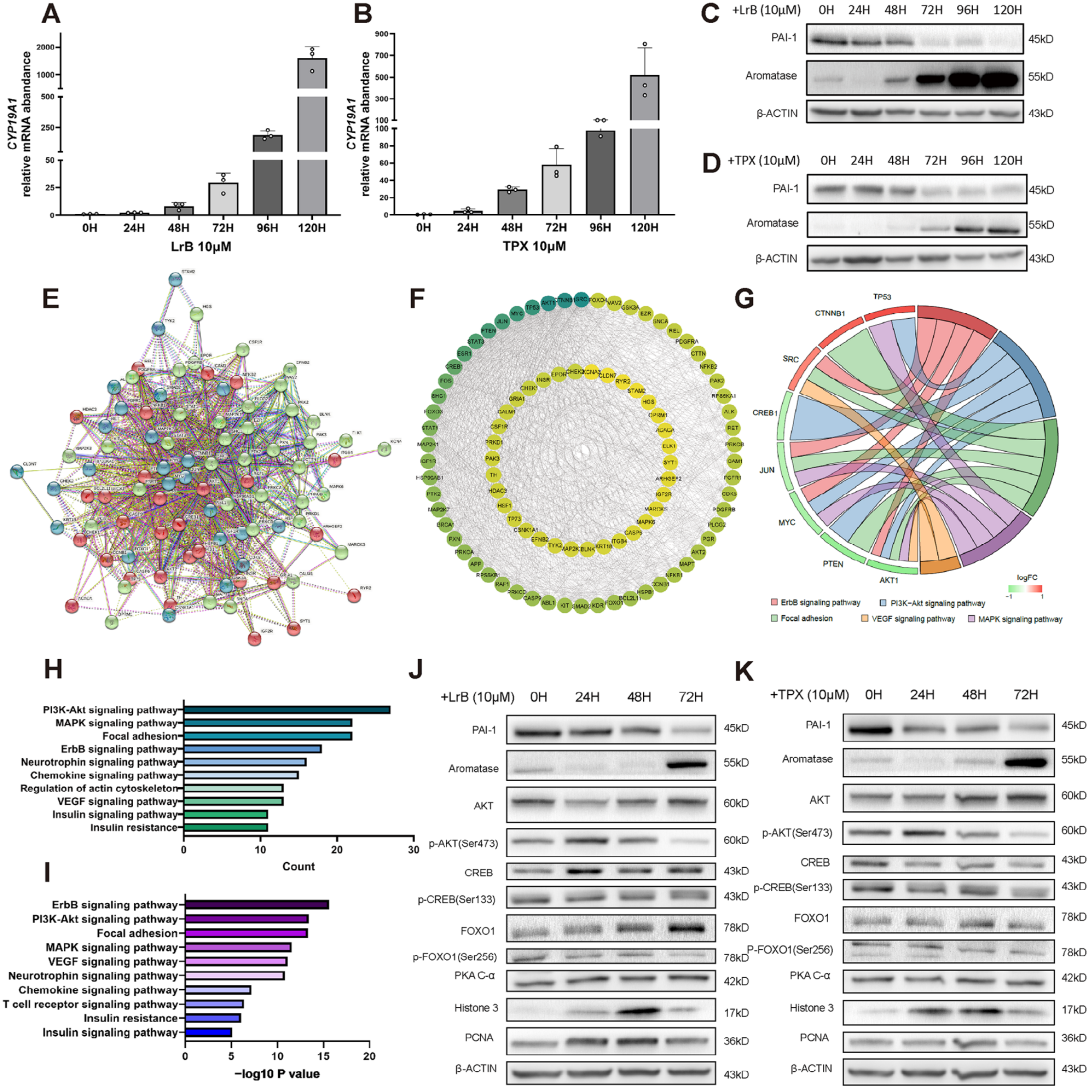
Participation of the PI3K/AKT pathway in the suppression of aromatase by SERPINE1. (A and B) qPCR analysis of CYP19A1 expression after treatment with Lrb (A, Loureirin B, 10 µM) or TPX (B, tiplaxtinin, 10 µM) for the indicated times in KGN cells (*n* = 3). (C and D) Western blot analysis of protein levels of PAI‐1 and aromatase following treatment with Lrb (C, 10 µM) or TPX (D, 10 µM) for the indicated times in KGN cells. β‐ACTIN was used as a loading control (*n* = 3). (E) Phospho‐antibody array to detect changes in signaling pathway phosphorylation and protein expression levels at 0 and 15 min after the addition of TPX (10 µM) to KGN cells. STRING analysis of differentially phosphorylated proteins (FC ≥ 1.5) was performed to predict the interactions with proteins related to PI3K–Akt signaling pathway (red), MAPK signaling pathway (blue) and Ras signaling pathway (green). (F) Protein interaction network (PPI) of differentially phosphorylated proteins in phospho‐antibody array. The deeper the green color, the stronger the interactions of the nodes. The top 10 genes are SRC, CTNNB1, AKT1, TP53, MYC, JUN, PTEN, STAT3, ESR1, and CREB1. (G) Chordal graphs showing the alignment of the top ranked genes with KEGG analyses. (H and I) KEGG analysis on the differentially phosphorylated proteins. The number of enriched proteins (H) and the significance‐log10 *p* value (I) are indicated, respectively. (J and K) Western blot analysis of expression levels of proteins related to AKT signaling pathway, CREB signaling pathway, FOXO1 signaling pathway, and PAI‐1 at indicated time points after the addition of LrB (J, 10 µM) or TPX (K, 10 µM) in KGN cells. β‐ACTIN was used as a loading control (*n* = 3).

To further validate the hypothesis, we interfered KGN cells with two SERPINE1 inhibitors, Loureirin B (LrB) and tiplaxtinin (TPX). qPCR results indicated a 10‐fold sharp increase in *CYP19A1* expression after 72 h of LrB treatment (Figure 5A) or a threefold increase with TPX (Figure 5B). Western blotting results supported this phenomenon, with a decreasing level of PAI‐1 and an abrupt surge of aromatase expression at 72 h (Figure 5C,D). Cell Count Kit‐8 experiments showed that the inhibitors had no effects on cell proliferation during this process (Figure S7A). Then, we used Phospho Explorer Antibody microarray (PEX100) to analyze signaling pathways protein changes at 0 and 15 min of TPX treatment. Differentially phosphorylated proteins were divided into three clusters according to the following pathways: AKT, Ras, and MAPK (Figure 5E). We used protein interaction network (PPI) networks and Metascape to identify protein interactions and Cytoscape to visualize them (Figure 5F). Functional enrichment analysis from high‐degree proteins and the top five pathways revealed that AKT1 had a high degree of protein interactions and the PI3K/AKT pathway had the highest number of interacting proteins (Figure 5G). Meanwhile, KEGG and pathway mapping of differentially phosphorylated proteins revealed significant PI3K/AKT pathway enrichment (Figures 5H,I and S7B). For further confirmation, we conducted western blotting and found that AKT were significantly phosphorylated after 15 min of TPX treatment (Figure S7C). When two different inhibitors were added for 72 h, phosphorylated AKT showed a similar pattern, increasing in the first 48 h and then decreasing (Figures 5J,K and S8). These findings suggest that PI3K/AKT plays a pivotal role in the downregulation of aromatase caused by elevated SERPINE1 level.

### 2.5 TPX Effectively Improves Disorders Caused by Dehydroepiandrosterone and Continuous Darkness in Rats

**FIGURE 6:**
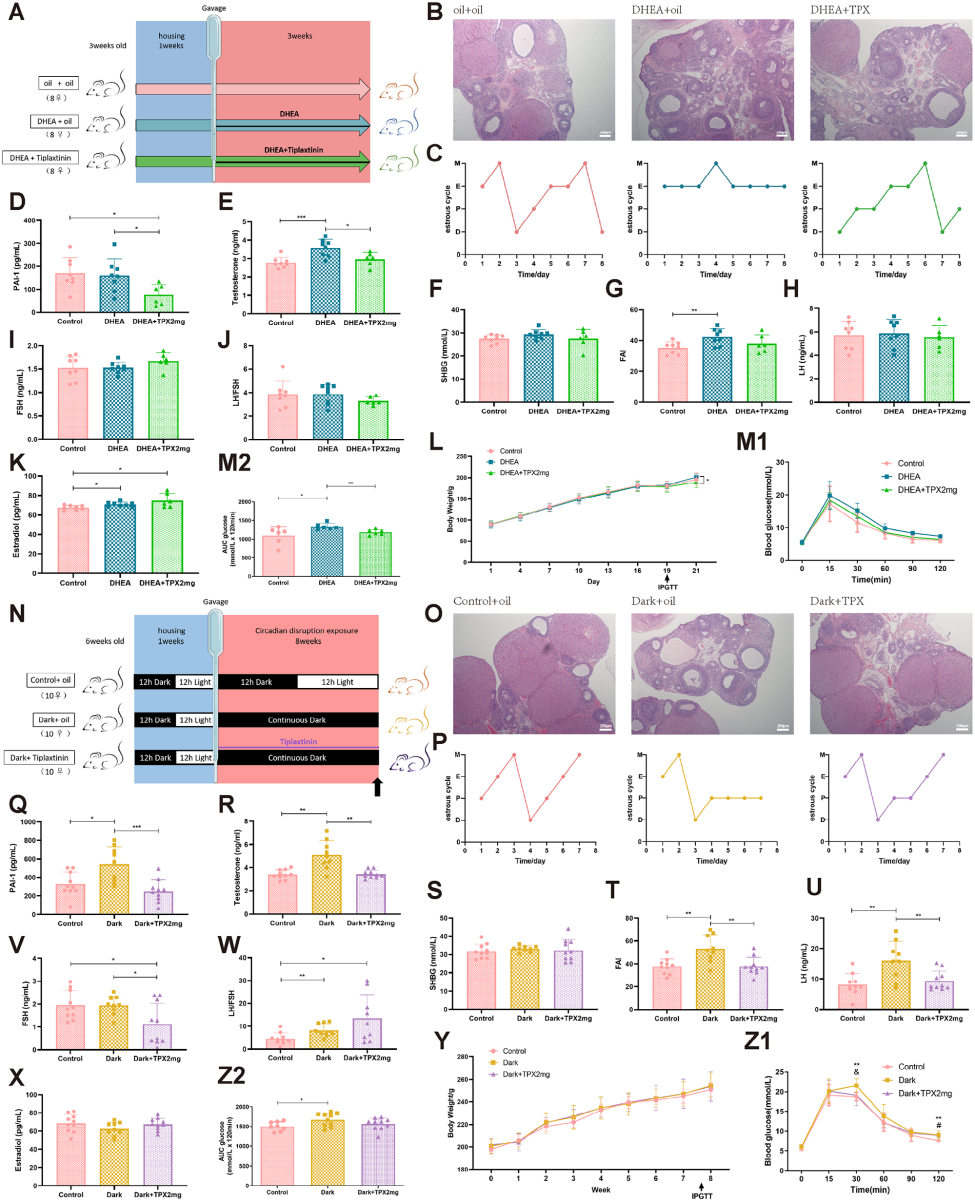
PAI‐1 inhibitor tiplaxtinin effectively ameliorates endocrine disorders and abnormal glucose metabolism in arrhythmic rats and DHEA rats. (A) Schematic illustration of dehydroepiandrosterone (DHEA) modeling and TPX (2 mg/kg) treated modeling design. (B) Representative images of H&E staining of ovaries from controls (oil + oil), DHEA group (DHEA + oil) and DHEA combined TPX treatment group (DHEA + TPX), scale bars, 200 µm. (C) Representative estrous cycles of 8 rat/group during 8 consecutive days, which were detected using a vaginal smear. M: metestrus, E: estrus, P: proestrus, D: diestrus phase. (D–K) Levels of PAI‐1 (D), testosterone (E), SHBG (F), LH (H), FSH (I), estradiol (K) in rat serum were measured using ELISA. FAI (G) is calculated as FAI = T/SHBG × 100%. LH/FSH ratio (J) is calculated as LH divided by FSH (control: *n* = 8; DHEA: *n* = 8; DHEA + TPX: *n* = 6). (L) Body weight curves of DHEA modeling and TPX (2 mg/kg) treated modeling rats during 21 days (control: *n* = 8; DHEA: *n* = 8; DHEA + TPX: *n* = 6). (M) The IPGTT of rats in three groups (M1) and the corresponding glucose area under the curve (AUC, M2) during the IPGTT (control: *n* = 6; DHEA: *n* = 6; DHEA + TPX: *n* = 6). (N) Schematic illustration of continuous dark modeling and TPX (2 mg/kg) treated modeling design. (O) Representative images of H&E staining of ovaries from controls (control + oil), dark group (dark + oil), and dark group with TPX treatment (dark + TPX), scale bars, 200 µm. (P) Representative estrous cycles of 8 rat/group during 8 consecutive days, which were detected using a vaginal smear. M: metestrus, E: estrus, P: proestrus, D: diestrus phase. (Q–X) Levels of PAI‐1 (Q), testosterone (R), SHBG (S), LH (U), FSH (V), estradiol (X) in rat serum were measured using ELISA. FAI (T) is calculated as FAI = T/SHBG × 100%. LH/FSH ratio (W) is calculated as LH divided by FSH (control: *n* = 10; dark: *n* = 9; dark + TPX: *n* = 10). (Y) Body weight curves of continuous dark modeling and TPX (2 mg/kg) treated modeling rats during 8 weeks (control: *n* = 8; dark: *n* = 8; dark + TPX: *n* = 9). (Z) The IPGTT of rats in three groups (Z1) and the corresponding glucose area under the curve (AUC, Z2) during the IPGTT (control: *n* = 8; dark: *n* = 10; dark + TPX: *n* = 10). Data are shown as mean ± SD. *p < 0.05, **p < 0.01, ***p < 0.001 (one‐way ANOVA).

As we previously observed hypoandrogenic effects after SERPINE1 inhibition, we investigated the therapeutic potential of SERPINE1 inhibitors in a preclinical study. We first tested the effect of simultaneous gavage of TPX or LrB at different concentrations for 21 days during dehydroepiandrosterone (DHEA) modeling (Figures 6A and S9A). Compared with controls, the DHEA‐treated rats exhibited impaired ovulation, as evidenced by follicles mostly blocked in the preantral follicular phase and stalled and disturbed estrous cycles. Notably, TPX 2 mg/kg, rather than TPX 20 mg/kg or LrB, effectively reduced the number of preantral follicles, with increasing the number of corpus luteum and restoring normal cycles (Figures 6B,C and S9B). TPX treatment effectively suppressed PAI‐1 levels (Figure 6D) and restored abnormal testosterone and FAI levels in DHEA‐treated rats in the absence of significant SHBG and gonadotropin fluctuations (Figure 6E–J). Notably, the PAI‐1 level did not significantly decrease in the DHEA‐treated group, leading to the speculation that SERPINE1 might serve as an upper stimulus, instead of a response, for elevated androgen level in vivo (Figure 6D). In line with the high androgen levels, the aromatase‐promoting effect of TPX induced a slight increase in E2 levels (Figure 6K). Furthermore, the body weight of the rats decreased with TPX 2 mg/kg (Figures 6L and S9C). The IPGTT results indicated that the glucose metabolic effect of TPX 2 and 20 mg/kg was significantly more potent than that of LrB at any dose (Figures 6M and S9D,E). These findings indicate that PAI‐1 plays a therapeutic role in hyperandrogenism‐induced PCOS.

Next, we administered continuous TPX treatment via gavage to the rats during the 8‐week continuous darkness exposure (Figure 6N). Consistent with previous findings, the dark group exhibited manifestations of ovarian morphology, estrous cycles, and elevated PAI‐1 levels, as previously observed (Figure 6O–Q). TPX significantly decreased the PAI‐1 levels and the androgen levels (Figure 6Q–T), further validating the downregulatory effect of SERPINE1 on androgens in vivo. As expected, the LH level elevation in the dark group was significantly reversed by TPX, but the FSH levels decreased after TPX treatment (Figure 6U–W), suggesting that TPX exerts a potential suppressive effect on gonadotropin secretion. Interestingly, contrary to the DHEA models, we did not observe a similar accumulation of E2 levels (Figure 6X). Although the body weight of rats did not change (Figure 6Y), the TPX treatment partially rescued darkness‐induced abnormalities in glucose metabolism (Figure 6Z). These results indicate the therapeutic effect of PAI‐1 in circadian disruption‐induced hyperandrogenism.

### 2.6 SERPINE1 is Hypomethylated and Overexpressed in Women with PCOS

**FIGURE 7:**
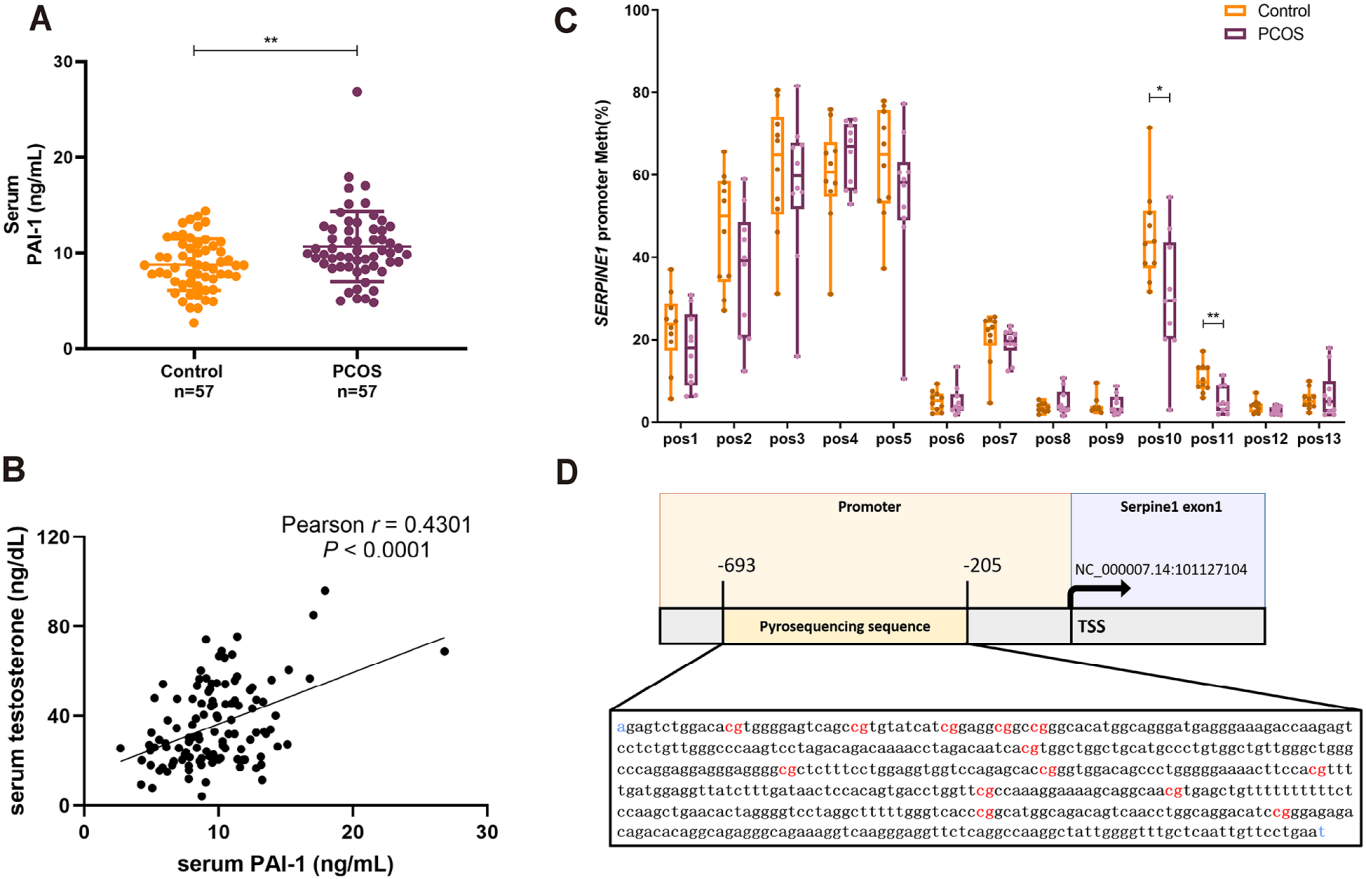
SERPINE1 is upregulated and hypomethylated in women with PCOS. (A) ELISA array of serum PAI‐1 levels from a cohort including 57 PCOS patients and 57 controls (*p* = 0.0021). (B) Pearson analysis between serum testosterone and PAI‐1 levels from 57 patients with PCOS and 57 controls (*n* = 114, Pearson *r* = 0.4301, *p* < 0.0001). (C) Pyrosequencing to detect methylation status of SERPINE1 promoter CpG sites of peripheral leukocytes in a cohort including 10 PCOS patients and 10 controls from the cohort in (A). Pos, CpG site in SERPINE1 promoter; Meth, methylation level. The positions of 13 CpG sites are shown in (D). (D) Schematic illustration of pyrosequencing sequences in patients. Data are shown as mean ± SD. Student's *t* test in (A) and (C), Pearson *r* test in (B). *p < 0.05, **p < 0.01, ***p < 0.001.

To determine whether our PCOS findings in rats might be associated with those in humans, we first confirmed the serum PAI‐1 level in a cohort of 57 women with PCOS and 57 controls. As a result, the PAI‐1 levels were significantly elevated in those with PCOS (**Figure** **7A** **and** **Table** **S1**). In addition, we characterized a significant positive correlation between serum PAI‐1 and testosterone levels in this cohort (Figure 7B). PAI‐1 also had a strong correlation with BMI and HOMA‐IR (Figure S10A,B), as reported in previous literature [28, 29]. But we found no difference in PAI‐1 in follicular fluid from patients with PCOS and controls (Figure S10C and Table S2). Consistent with data from rat GCs, the methylation level of CpG in the *SERPINE1* promoter decreased in the peripheral leukocytes of patients with PCOS (Figure 7C,D and Table S3). Notably, the two CpG sites (positions 10 and 11) that underwent significant changes were located in the promoter region close to the TSS, which was very similar to the changes observed in the rats.

## 3 Discussion

Environmental and epigenetic mechanisms play an important role in the etiology of PCOS. Although previous studies have identified a link between circadian disruption and PCOS development [6, 7, 8, 9, 10], the impact of the epigenome in this process has not been systematically investigated. In this study, we profiled DNA methylation via target‐captured methylation sequencing in female rat GCs using a longitudinally monitored continuous darkness model. For the first time, we have elucidated the underlying epigenetic epistasis of PCOS caused by circadian stimuli, elaborating on the process by which the *Serpine1* gene is hypomethylated and regulates androgen metabolism. At both cellular and physiological levels, we demonstrate the specific mechanism of androgen excess by *Serpine1* and the therapeutic effects on hyperandrogenism by SERPINE1 inhibitors. We propose SERPINE1 as a biomarker for clinical diagnosis and epigenetic‐based therapy in PCOS.

Current evidences indicate a mutually reinforcing relationship between circadian disruption and PCOS. On the one hand, excessive androgens during puberty disrupted the phase distribution of multiple circadian oscillators in the reproductive and metabolic axes in rats [30]. Interestingly, excessive androgens can reduce H3K27me3a by inhibiting the expression of histone methyltransferase, Ezh2, and increase gene silencing of circadian genes via an epigenetic pathway [31]. On the other hand, chronodisruption in ovarian GCs of PCOS [7] may cause hyperandrogenism. Recent studies show that women with PCOS have higher percentages of evening chronotypes, which means an increased risk of developing sleep disturbances, and have elevated and delayed nocturnal and morning melatonin levels. Both evening chronotypes [8] and delayed melatonin levels [10] are tightly positively associated with high testosterone levels. The above epidemiological data and studies in model organisms suggest a correlation between circadian disruption and hyperandrogenism in PCOS but failed to draw causal conclusions. Therefore, we addressed whether and how a PCOS‐like phenotype could be induced by circadian disruption. Our previous studies comparing continuous light and continuous darkness [12] found that rats exposed to 8‐week darkness exhibited more violent disruption in core‐clock gene expression and more severe PCOS‐like manifestation characterized than continuous light exposure. These manifestations included elevated serum testosterone, irregular menstrual cycles, polycystic ovarian morphology, and metabolic disturbances such as insulin resistance, which are clinically relevant to human PCOS. Therefore, we selected the continuous darkness model as a preclinical model to explore the mechanisms underlying the etiology of the disease. It is worth mentioning that rats, as nocturnal animals, in contrast to human circadian behavior, are awake and active at night and in a static sleep state during the day. Therefore, continuous darkness actually mimics extreme prolongation of waking hours in humans, such as night owls and all‐nighters, which are common nowadays.

Epigenetic modifications, which are labile circadian interventions, can dynamically adjust transcriptional networks without altering the genetic material. DNA methylation is the most classic epigenetic modification in mammals, and its level within the promoter region to the first exon is generally associated with transcriptional repression [32]. DNA methylation dynamics have been shown to occur circadian [33]. Combining different circadian models and human genome‐wide studies, circadian‐related methylation changes have been found to be heterogeneous, including both hyper‐ and hypomethylation at specific genomic locations between different tissues [34, 35]. Although the longitudinal global DNA methylation in our study was highly conserved, the results indicated that different methylation and expression genes were implicated in the regulation of signaling, neuroactive ligand–receptor interaction, hormone activity, one‐carbon pool by folate, fatty acid biosynthesis, antigen processing, and immune system, consistent with several clinical studies reporting DNA methylation changes relevant to lipid metabolism, inflammation, and neurological and endocrine systems in women with PCOS [36, 37].

We used Agilent SureSelectXT MC‐seq capture sequencing. The system focuses on DMRs that affect gene expression, including GENCODE promoters, CpG islands, CpG island shores, CpG island shelves, and Dnase1 high‐sensitivity sites [38]. In our sequencing, each sample provided approximately 20 G of raw data, with a sequencing depth of >10× and targeted capture region of 90‐Mb segment of the rat genome. The target read ratio fluctuated from 71.27 to 84.26%. Compared with methylation beads, it identified significantly more CpG sites than the 385 K promoter sites in the rat methylation beads. Furthermore, MC‐seq identified regions that could not be detected by RRBS. Despite whole‐genome bisulfite sequencing (WGBS) being the gold standard for methylation sequencing, the DNA extracted from the GCs of a single rat ovary are insufficient to satisfy the WGBS library construction criteria due to large genomic DNA input to compensate for degradation during DNA bisulfite treatment. In summary, MC‐seq enables profiling of significantly more CpG sites than methylation beads, requires less genomic DNA input than WGBS, and improves throughput.

On the basis of the strong association between *Serpine1* and androgens, we decided to investigate it in depth. As aforementioned, *Serpine1* is a glycoprotein that modulates extracellular matrix remodeling and fibrinolysis and is elevated in patients with PCOS but is more associated with BMI. Mechanistically, we observed significant hypomethylation in the *Serpine1* promoter and verified using 5‐Aza treatment and CRISPR–dCas9‐based targeted methylation that hypomethylation near the TSS of the *Serpine1* gene unequivocally causes gene overexpression. Interestingly, we found that the DNA methylation signatures and associated gene expression alterations detected in the GCs of the dark group were also present in the liver, a metabolic tissue. These consistent changes indicate that such methylation landscape and transcriptional changes were not incidental hypomethylation events in individual organs but rather systemic events in which different organs make the same changes in response to the same environmental alterations. Furthermore, diurnal PAI‐1 gene expression is thought to be directly driven by binding its promoter region to clock gene proteins such as CLOCK and BMAL1/BMAL2 [39]. In diurnal humans and nocturnal rodents, plasma PAI‐1 levels were elevated at the onset of the active phase, suggesting that rhythmic PAI‐1 expression is necessary for the organism. Although we did not observe methylation changes at the CpG site at the CLOCK/BMAL1 binding site in the *Serpine1* promoter region, the binding of the CLOCK/BMAL1 complex to PAI‐1 may be altered by chromatin conformational changes. These interesting speculations deserve to be tested in more experiments.

We performed pharmacological inhibition of PAI‐1 with TPX in KGN cells and then confirmed our results in continuous darkness and DHEA‐treated rats. TPX significantly decreased serum PAI‐1 levels and reduced abnormally high testosterone levels in both continuous darkness‐exposed rats and DHEA‐treated rats. To the best of our knowledge, this is the first evidence of the therapeutic potential of PAI‐1 for hyperandrogenism in PCOS in a preclinical model. PAI‐1 also attenuated polycystic ovarian morphology and rectified estrous cycles. The protective effect of TPX was also observed in deficient glucose metabolism. TPX, one of the most studied PAI‐1 inhibitors, appears to be a very promising drug. The in vivo efficacy of TPX has been demonstrated in several animal models [40]. It does not have side effects, such as bleeding time, thrombin time, or blood pressure, and has high oral bioavailability [40]. Other inhibitors derived from this inhibitor structure (e.g., PAI‐749) were investigated in human phase I clinical trials of fibrinolysis [41]. Additional preclinical and clinical trials on TPX for hyperandrogenism should be conducted.

We observed SERPINE1 hypomethylation and overexpression in women with PCOS. Notably, with age and BMI matching in these cohorts, we found a significant positive correlation between serum PAI‐1 and testosterone levels. These findings support the findings in our continuous darkness‐exposed rat model, and provide a rationale for early identification of potential PCOS based on blood SERPINE1 methylation or expression status. Although this study was limited by a small sample size, it initially validated our findings in the real world. Therefore, a larger clinical study is necessary.

There are some limitations to this study. The opposite chronobiological behavior of the rat to that of humans cannot be ignored, especially about insurmountable discrepancies in simulating light reflection, melatonin secretion, metabolism, and social behavior [42]. In the future, diurnal rodents [43] or nonhuman primate [44] chronological models should be developed for further study. Furthermore, we focused on the PI3K/AKT pathway to investigate the potential mechanism by which PAI‐1 inhibits aromatase expression. However, the antibody microarray showed that alternative mechanisms contributing to this inhibition may still be possible, which require further investigation.

As the current PCOS treatment is symptom‐based and associated with high recurrence rate, there is an urgent need to develop new therapeutic products that can address the initiation and pathogenetic mechanisms of the syndrome. This study demonstrated that the differentially methylated gene *Serpine1* detected in the GCs of continuous darkness‐exposed rats is also altered in blood samples from women with PCOS compared with controls, with concomitant upregulated expression and related hyperandrogenism. This exciting finding provides new avenues for the early detection of susceptibility to PCOS and candidate biomarkers for epigenetic‐based therapies. Future research is warranted to identify the markers in larger cohorts of patients and epigenetic editing of SERPINE1 in animal models so as to assess epigenetic therapeutic effects in vivo.

## 4 Methods

### 4.1 Animal Use and Care

The animal study protocols were approved by the Animal Ethics Committee of Shanghai Model Organisms Center (2021‐0011‐01) and Shanghai University of Traditional Chinese Medicine (PZSHUTCM211101047). All animal experiments were carried out in accordance with the guidelines of the local animal ethics committee.

### 4.2 Continuous Darkness‐Exposed Rat Model

The continuous‐darkness‐exposed SD rat model has been generated as previously described [11, 12]. A total of 80 female SD rats (5–6 weeks) were used after 1 week of a 12:12 h light–dark cycle acclimatization. The rats were randomly divided into two groups: (1) the dark group, caged with impermeable black cage cloth, resulting in a continuous dark environment and (2) the control group, kept under a 12:12 h light–dark cycle. Eight rats from each group were sacrificed every 2 weeks for total 8 weeks.

### 4.3 DHEA Treatment

Immature (21 d old) female SD rats were injected daily with DHEA (60 mg kg^−1^/d, dissolved in 0.2 mL of sesame oil, D4000; Sigma Aldrich, St. Louis, USA) subcutaneously, as the DHEA group. The control group received injections of the same volume of sesame oil daily. The above animals were all treated for 3 weeks.

### 4.4 TPX Treatment

TPX was administered by gavage as a rescue treatment. For the darkness‐treated model, there were three groups: (1) dark + TPX, kept under a continuous dark environment and gavaged with TPX (2 mg kg^−1^/d, diluted in 1% DMSO in corn oil, HY‐15253; MedChemExpress, New Jersey, USA), (2) dark + oil, kept under a continuous dark environment and gavaged with corn oil (HY‐Y1888; MedChemExpress), (3) control + oil, kept under a 12:12 h light–dark cycle and gavaged with corn oil. The above animals were all treated for 8 weeks. For the DHEA‐treated model, there were three groups: (1) DHEA + TPX, injected with DHEA (60 mg kg^−1^/d) and gavaged with TPX (2 mg kg^−1^/d), (2) DHEA + oil, injected with DHEA (60 mg kg^−1^/d) and gavaged with corn oil, (3) oil + oil, injected with sesame oil and gavaged with corn oil. The above animals were all treated for 3 weeks.

### 4.5 MC‐seq DNA Methylation Data Analysis

For MC‐seq, raw image files obtained from sequencing were identified by base identification and error filtering to obtain raw sequencing fragments that could be used for analysis, and the results were stored in FASTQ file format. Sequence data quality was examined using FastQC (ver. 0.11.5). The bases with low quality scores and the adapters in all the sequenced reads were removed by Trim_galore (ver. 0.4.1). The trimmed reads were mapped to Rattus norregicus genome (RAT6.0, ensemble) by Bismark pipelines (ver. v0.15.0; bowtie v2.2.9). Duplicated reads were removed by deduplicate_bismark. All CpG sites with coverage ≥10× depth were retained for analysis to ensure high MC‐Seq data quality. Each sample provides approximately 20 G of raw data after sequencing, with a targeted capture area of approximately 90 Mb. The target read ratio varies from 71.27 to 84.26%, and the threshold value was set as: *p* < 0.05, methylation difference degree >10%. The DMRs were obtained by the R package DSS call DMR function with the DMR length (less than 1000 bp) and CpG number (at least 3 CpGs in a DMR), and the threshold value was set as: pairwise Chi‐squared tests with subsequent multiple testing correction; adjusted *p* < 0.05, a minimal difference cutoff of 10%. The promoter region is defined as the regions from −2.5 to +0.5 kb of the tTSSs. The selected DMS‐associated genes were mapped to each term in the GO database, the KEGG database and the Reactome database using KOBAS‐i (KOBAS 3.0), and the number of genes in each term was calculated. Fisher's exact test was used to measure the gene‐enrichment in annotation terms. Principal component analysis was performed using R (v.3.3.1).

### 4.6 RNA‐seq Data Analysis

The raw pair‐end RNA‐seq FASTQ data were trimmed to remove adaptor sequences and low‐quality bases by Seqtk (GitHub—lh3/seqtk: Toolkit for processing sequences in FASTA/Q formats) with default settings. The trimmed reads were mapped to Rattus norregicus genome (RAT6.0, ensemble) using Hisat2 (version:2.0.4). The expression level of each gene was calculated as fragments per kilobase of exon model per million mapped reads (FPKM) by Stringtie (version:1.3.0) and TMM (trimmed mean of M values). DEGs analysis was performed by using edgeR. The DEG were as follows: at least 1.5‐fold change and adjusted *p* value (attained by the Wald test and corrected for multiple testing using the Benjamini–Hochberg method by default) less than 0.05. The DEGs were submitted to the DAVID 6.8 website to perform KEGG analysis, and Fisher's Exact test was used to measure the gene enrichment.

### 4.7 Correlation Analysis of Methylation and Transcription

To obtain correlation between differential methylation and gene expression, we considered only DMSs in promoter regions. We plotted log2‐fold expression changes versus methylation differences, and the distribution of genes among the four resulting quadrants was tested for directionality using Fisher's exact test.

### 4.8 CRISPR–dCas9‐Based Targeted Methylation in BRL‐3A Cells

The sgRNA–ZsGreen1–T2A–Puro (PGMLV‐SpCas9) vectors (GM‐8199) and GPLVX–CMV–3×Flag–NLS–dCas9–Dnmt3aCD–T2A–Blasticidin vectors (10948) were purchased from Genomeditech (Shanghai, China). To generate stable cell lines with integrated dCas9–Dnmt3a transgenes, the BRL‐3A cells were seeded into a six‐well plate and transfected the next day at 30–50% confluence. We added 5 µg/mL polybrene (Sigma–Aldrich) to 10% FBS DMEM. BRL‐3A cells were transduced with lentiviral particles of GPLVX–CMV–3×Flag–NLS–dCas9–Dnmt3aCD–T2A–Blasticidin according to the provider's protocol. Stably integrated cells were selected with blasticidin S (25 µg/mL, GM‐040404; Genomeditech) for 48 h. Then stable dCas9–Dnmt3aCD–BRL–3A cells were transduced with lentiviral particles of different sgRNA–ZsGreen1–T2A–Puro vectors separately (Table S4). Stably integrated cells were selected with puromycin (1.5 µg/mL, GM‐040401; Genomeditech). Cells were harvested 3 days postinfection in this study.

### 4.9 Phospho‐Antibody Array Analysis

Identification of PAI‐1 targeting proteins was performed by phospho‐antibody array (Full Moon, Sunnyvale, USA) analyzed with phospho‐Exploerer (PEX100) by Wayen Biotechnologies (Shanghai, China) using KGN cells. KGN cells were treated with TPX (10 µM) for 15 min, then the cells were collected for the array. The phosphorylation signal ratio was calculated as follows: phospho‐ratio = phospho/unphospho. The ratio of the phospho‐ratio between 15 and 0 min was calculated as follows: ratio of phospho‐ratio = phospho‐ratio (15 min)/phospho‐ratio (0 min). The Fold Change threshold was set to 1.5 to obtain the differentially phosphorylated proteins.

### 4.10 Clinical Samples

This study enrolled patients who underwent in vitro fertilization‐embryo transfer treatment at Department of Reproductive Medicine in Ren Ji Hospital Affiliated to Shanghai Jiao Tong University School of Medicine from January 2015 to December 2017. Discarded serum, plasma and follicular fluid from patients were collected for the study. The Medical Ethics Committee of Ren Ji Hospital Affiliated to Shanghai Jiao Tong University School of Medicine approved this study process (no. 2017041411), and all participants provided written informed consent. The main reference for the diagnostic criteria of PCOS was the Rotterdam Consensus for PCOS revised in 2003. Basic and PCOS‐related anthropometric variables are presented in Tables S1–S3.

### 4.11 Statistical Analysis

All data are presented as mean ± SD. Each experiment/group used 3–5 samples/independent replicates. Statistical analyses were conducted using IBM SPSS Statistics and GraphPad Prism 8 (GraphPad Software). Statistical significance was analyzed using Levene's test for equality of variances. Differences between two groups were determined by two‐tailed Student's *t* test where data was normally distributed data. Differences between more than two groups were determined by one‐way ANOVA followed by Dunnett's post hoc test or the Kruskal–Wallis test followed by Dunn's post hoc test. The correlation between the two data sets was analyzed using the Pearson correlation coefficient when equal variance was assumed. *p* < 0.05 was considered as statistically significant.

## Author Contributions

**Xueying Geng**: conceptualization, formal analysis, methodology, investigation, software, visualization, funding acquisition, writing—original draft. **Weiwei Chu**: formal analysis, methodology, investigation, validation, writing—review and editing. **Shang Li**: data curation, investigation, writing—review and editing. **Xiying Zhou**: investigation, methodology, writing—review and editing. **Dongshuang Wang**: data curation, investigation, writing—review and editing. **Junyu Zhai**: data curation, investigation, writing—review and editing. **Yun Sun**: data curation, project administration, funding acquisition, writing—review and editing. **Zi‐Jiang Chen**: conceptualization, funding acquisition, writing—review and editing. **Yanzhi Du**: conceptualization, funding acquisition, project administration, writing—review and editing. All authors have read and approved the final manuscript.

## Conflicts of Interest

The authors declare no conflicts of interest.

## Ethics Statement

The animal study protocols were approved by the Animal Ethics Committee of Shanghai Model Organisms Center (2021‐0011‐01) and Shanghai University of Traditional Chinese Medicine (PZSHUTCM211101047). All animal experiments were carried out in accordance with the guidelines of the local animal ethics committee.

The clinical sample collections were approved by the Medical Ethics Committee of Ren Ji Hospital Affiliated to Shanghai Jiao Tong University School of Medicine (no. 2017041411), and all participants provided written informed consent.

## Supporting information



Supporting Information

## Data Availability

The methylation data and transcriptomic data generated in this study are deposited in the Genome Sequence Archive in National Genomics Data Center, China National Center for Bioinformation / Beijing Institute of Genomics, Chinese Academy of Sciences (GSA: CRA014285, CRA014335) that are publicly accessible at https://ngdc.cncb.ac.cn/gsa. Other data generated or analyzed during this study are included in this article and its supplementary files.
