# Supplementary material for: Hypomethylation‐Triggered SERPINE1 (Serpin Family E Member 1) Exacerbates Polycystic Ovary Syndrome with Hyperandrogenism Induced by Circadian Disruption
Source: MedComm (2020). 2025 Jul 4;6(7):e70270. doi: 10.1002/mco2.70270 (PMC12231203; doi:10.1002/mco2.70270)
Supplement: Supplementary file 1 — Supporting Information [file MCO2-6-e70270-s001.pdf]

Supplementary material for

Hypomethylation-triggered SERPINE1 (serpin family E member 1) exacerbates polycystic ovary syndrome with hyperandrogenism induced by circadian disruption

Xueying Geng[1,2], Weiwei Chu[1,2], Shang Li[1,2], Xiying Zhou[1,2], Dongshuang Wang[1,2], Junyu Zhai[1,2], Yun Sun[1,2], Zi-Jiang Chen[1,2,3*] and Yanzhi Du[1,2*]

*Correspondence: chenzijiang@hotmail.com (Z.C); duyz@sjtu.edu.cn (Y.D.)

This PDF file includes:

Supplemental Materials and Methods

Figures S1 to S10

Tables S1 to S12

Supplemental Materials and Methods

Animal use and care

All Sprague Dawley (SD) rats (Charles River Laboratories, Shanghai, China) were housed 3–5 per cage under specific pathogen-free conditions in a 12-h light-dark cycle with temperature range of 21°C ± 2°C and humidity range of 50% ± 5%. Rats had ad libitum access to water and food. All rats were treated after arrival into the animal facility and after an acclimatization period of at least 1 weeks. Rats were randomly divided to groups at the time of arrival to minimize any potential bias. The sample size, sex and age of the animals used is specified in the text and/or figure legends. At finalization, mice were anesthetized with isoflurane, and blood was collected through the abdominal aorta. The ovaries, oviduct ampulla, adipose tissue, lung, spleen, kidney, liver and muscle samples were quickly dissected on ice, snap frozen and stored at -80°C or stored at 4% paraformaldehyde (Servicebio, Wuhan, China).

Assessment of reproductive phenotype

Body weight development was recorded weekly. The specific stage of the estrous cycle was assessed by daily vaginal cytology under an inverted microscope for 8 consecutive days. After the rats were sacrificed, the weights of bilateral intact ovarian tissue were obtained and recorded.

Histology

For hematoxylin and eosin (HE) staining, fresh ovaries were collected from model rats, fixed in 4% PFA solution and embedded in paraffin. Ovaries were cut through the maximal axis at 5 μm thickness and stained with hematoxylin-eosin (Cat # GHS132, HT1103128, Sigma Aldrich,

St. Louis., MO, USA). Sections were examined using a microscope (Zeiss, Oberkochen, Germany).

For immunohistochemistry (IHC), ovary sections were blocked with BSA, incubated with FSHR antibody (22665-1-AP, Proteintech Group, San Diego, CA, USA) at 1:200 dilution overnight at 4°C in a dark room and then incubated with the secondary antibody. Diaminobenzidine (DAB) was applied for the color reaction. All slides were imaged for 10 random fields at ×20 magnification using a microscope (Zeiss) and then analyzed using Image-Pro Plus 6.0.

Biochemical assessment of sex steroids and inflammatory factors

The fresh rat blood samples were centrifuged at 2500 rpm for 20 min at 4°C and stored at −80°C for subsequent serum assessments. The levels of testosterone (KGE010, R&D, Minnesota, USA), sex hormone-binding globulin (MBS014745, MyBiosource, San Diego, CA, USA), estradiol (KGE014, R&D), follicle stimulating hormone (EKU04249, Biomatik, Ontario, Canada), luteinizing hormone (MBS453439, MyBiosource), interleukin 1 beta (IL-1b) (MBS2023030, MyBiosource), interleukin 6 (IL-6) (BMS625, Invitrogen, Carlsbad, CA, USA), tumor necrosis factor-alpha (TNF-alpha) (MBS2507393, MyBiosource), and plasminogen activator inhibitor 1 (PAI-1) (CSB-E07948r, Cusabio, Barksdale, DE, USA) were measured by enzyme-linked immunosorbent assay (ELISA) kits in serum of rats according to the manufacturers' instructions. Serum testosterone and SHBG were used to calculate the free androgen index (FAI) as (testosterone (nmoll−1) × 100) / SHBG (nmoll−1).

Assessment of metabolic phenotype

Fasting blood glucose levels were measured after a 14-h fast (starting from 6:00 PM) for rats. Blood glucose levels were determined in blood samples from the tail vein at 8:00 AM using an automatic glucometer (AccuCheck, Roche, Basel, Swiss). For intraperitoneal glucose tolerance test (ipGTT), rats were fasted for 14 h (starting from 6:00 PM) and injected intraperitoneally with 50% glucose water (2g per 1kg bodyweight). Blood glucose levels were measured in blood samples from the tail vein at time 0 (before glucose administration) and at 30, 60, 90 and 120 min after glucose administration using an automatic glucometer (AccuCheck, Roche). AUCs were used for evaluating the efficacy for the maintenance of glucose homeostasis.

The serum lipids—total cholesterol, triglycerides, high-density lipoprotein cholesterol (HDL-C), low-density lipoprotein cholesterol (LDL-C), and nonesterified fatty acid (NEFA) were determined using an autoanalyzer (AU5800, Beckman Coulter, CA, USA). The level of Leptin (MOB00B, R&D) was measured by ELISA kits in serum of rats according to the manufacturers' instructions.

Rat ovarian granulosa cells collection

To obtain granulosa cells, female SD rats were superovulated by intraperitoneal injection of pregnant mare's serum gonadotropin (PMSG; 20IU per 1kg bodyweight, 200IU/mL diluted in saline, SUMUYAOYE, Suzhou, China) followed by human chorionic gonadotropin (hCG; 20IU per 1kg bodyweight, 200IU/mL diluted in saline, SUMUYAOYE) 44-48 h later. Cumulus–oocyte complexes and superovulated ovaries were collected at 14-16 h after hCG injection respectively. For cell culture, superovulated ovaries were collected at 60 h after PMSG injection with no hCG injection. The cumulus cells were isolated and collected after brief incubation in 0.2% hyaluronidase (H3506, Sigma Aldrich). For mural granulosa cells collection, the ovary was

punctured with a 26-gauge needle and the granulosa cells were released in 1 ml of 1× PBS. The PBS containing the cells was filtrated through a cell strainer (70μm, 352350, BD Falcon, New Jersey, USA) and the filtrate was collected and centrifuged at 800 rpm for 5 min. The cells were resuspended using hyaluronidase (2mg/ml, H3506, Sigma Aldrich), placed at room temperature for 5 min, protected from light, and resuspended in culture medium after centrifugation at 800 rpm for 5 min, counted followed by cell culture or centrifuged again to collect granulosa cells for subsequent experiments.

Genomic DNA extraction

For low cell number samples, genomic DNA was extracted with a QIAamp DNA Mini Kit (51304, Qiagen, Dusseldorf, Germany) according to the manufacturer's instructions. For rats' tissues, genomic DNA was extracted with a TIANamp genomic DNA Kit (DP304, TIANGEN, Beijing, China) according to the manufacturer's instructions.

Library preparation for target-captured bisulfite sequencing

Rat ovarian granulosa cells extracted from animal experiments were used for DNA methylation capture sequencing assays (MC-seq). 1 μg of DNA calculated by Qubit was fragmented by sonication. Library preparation was performed with SureSelectXT Methyl Reagent kit (G9651A, Agilent Technologies, CA, USA) and Rat DNA methylation baits (931143, Agilent Technologies). The Methyl-Seq Kit could enrich rat genomic regions including CpG islands, CpG island shores, CpG island shelves, undermethylated sites, promoters and tissue-specific DMRs. DNA was bisulfite-treated using EZ DNA Methylation-Gold Kit (D5006, Zymo, Orange County, USA). Sequencing libraries were assessed by Agilent2100 Bioanalyzer and quantified with

Qubit® 2.0 Fluorometer. The libraries were then sequenced on HiSeq 2500 (2 × 101bp paired-end reads, Illumina, CA, USA).

Weighted gene co-expression network analysis (WGCNA)

The standard deviations of CpG loci were calculated, and the top8000 of them were taken to WGCNA. Weighted gene co-expression network analysis (WGCNA) (package in R) was used to identify the highly co-methylated-expressed gene modules associated with volatile contents. Determine the weighting factor β as 13, calculate the dissimilarity coefficient between methylation sites, cluster methylation sites by hierarchical clustering, and then use dynamic tree cut method to determine the gene module. The closer modules were merged into new modules by clustering analysis.

Short time-series expression miner (STEM) analysis of the DMSs

The Short Time Series Expression Miner (STEM) (v1.3.11) was used to provide an integrated solution to identify statistically significant time-dependent gene methylation and expression profiles (significant clustering was defined as $P < 0.05$). The significantly clustered DMSs showed a trend of gradual hyper- or hypo-methylation as one moved from the start to 8weeks of darkness and control using limma R package (version 3.46.0).

Pyrosequencing

Genomic DNA was bisulfite-converted with EZ DNA Methylation-Lighting™ Kit (D5031, Zymo). Bisulfite-treated DNA was amplified with Takara EpiTaq HS (Osaka, Japan). Primers for the pyrosequencing analysis were designed using PyroMark Assay Design software 2.0 (Qiagen),

and produced by Sangon Biotech (Shanghai, China). Pyrosequencing was carried out according to the manufacturer's standard protocol on a PyroMark Q48 instrument (Qiagen). All primer sequences are shown in **Table S5-S8**.

RNA extraction and purification

Rat tissues were homogenized in 1 ml RNAlater reagent (RL-01001, FOREGENE, Chengdu, China) with a Tissue Lyzer (BiHeng biotechnology Inc, Shanghai, China), and total RNA was isolated according to the manufacturer's instructions (RE-03011, FOREGENE). For low cell number samples, total RNA was extracted using RNeasy Mini Kit (74104, Qiagen) following the manufacturer's instructions. Qualified total RNA was further purified by RNAClean XP Kit (A63987, Beckman Coulter) and RNase-Free DNase Set (79254, Qiagen) for RNA sequencing.

Library preparation for RNA sequencing

Rat ovarian granulosa cells extracted from animal experiments were used for RNA sequencing (RNA-seq). 500 ng of total RNA (RNA integrity number ≥7) was assessed by Agilent Bioanalyzer 2100 (Agilent technologies) for library construction. RNA-seq libraries were generated using VAHTS Universal V6 RNA-seq Library Prep Kit for Illumina® (Vazyme, Nanjing, China) following the manufacturer's instructions. RNA-seq libraries were sequenced on Hiseq 2000 (paired-end reads, Illumina).

QPCR

For gene expression analyses, 1000ng of isolated RNA was reverse transcribed into cDNA using PrimeScript™ RT Master Mix (Takara) using the manufacturer's recommended cycling

conditions. QPCR was carried out using SYBR Premix Ex Taq (Takara). The 2-ΔΔCT method was applied to analyze mRNA expression levels normalized to housekeeping genes Beta-actin (ActB) levels. The primer sequences of the tested genes are listed in **Table S9-S10**.

Cell Culture and Treatments

The human GC line KGN cells were gifted from Prof. Toshihiko Yanase of Fukuoka University in Japan to Center for Reproductive Medicine, Shandong Provincial Hospital, Shandong University. KGN cells were cultured in Dulbecco's Modified Eagle's Medium/nutrient mixture F-12 (DMEM/F12) (Gibco, Waltham, Massachusetts, USA) containing 10% charcoal-stripped fetal bovine serum (FBS) (Thermo Fisher Scientific, Waltham, Massachusetts, USA) and 1% antibiotics (mixture of penicillin, streptomycin, and neomycin; Gibco) in a 37°C, 5% CO2 incubator (Thermo Fisher Scientific).

Rat granulosa cell was obtained as described above. After granulosa cells were isolated and collected by brief centrifugation. Cell viability and cell counting were determined by trypan blue staining. The granulosa cells were then cultured in the conditions as same as those of KGN cells.

Rat normal liver cell BRL-3A was purchased from the Cell Bank of the Shanghai Institutes for Biological Sciences, Chinese Academy of Sciences (GNR10, Shanghai, China). Cells were cultured in DMEM supplemented with 10% FBS (Thermo Fisher Scientific) and 1% PSN.

Cells were passaged every 2-3 days. KGN cells were authenticated via short tandem repeat PCR profiling, and no cross-contamination by other cells was observed in June 2018. BRL-3A cells were authenticated via species identification in February 2021. Rat GCs' cell purity and identity were identified by FSHR immunohistochemistry.

For 5-Aza treatment, cells were seeded into six-well plates. Twenty-four hours later, cells were washed and replaced with fresh medium containing 5-Aza (5μM or 10μM, A3656, Sigma Aldrich). Then, cells were collected after 48 or 72 h for use. For TPX treatment, cells were treated with TPX for 0-72 h (10μM, HY-15253, MedChemExpress, New Jersey, USA) before collection. For loureirin B (LrB) treatment, cells were treated with LrB for 0-72 h (10μM, HY-N1504, MedChemExpress) before collection.

Cell Transfection

The SERPINE1 (NM_001386460.1, Homo sapiens) sequence was cloned into pCMV-FLAG-HIS plasmids (TranSheepBio, Shanghai, China) to overexpress SERPINE1. The Serpine1 (NM_012620.1, Rattus norvegicus) sequence was cloned into pTSB02-GFP-PURO plasmids (TranSheepBio) to overexpress Serpine1. Three independent siRNAs against SERPINE1 or Serpine1 (**Table S11**) were obtained from Biomics (Nantong, China). Plasmids and/or siRNAs were transfected into KGN cells using Lipofectamine 3000 (Invitrogen) or into rat GCs using Lipofectamine RNAiMAX (Invitrogen) according to the manufacturer's instructions. After transduction, cells were cultured for 48 or 72 h before further treatment

Protein extraction and immunoblotting

Tissues were lysed in ice-cold radioimmunoprecipitation assay lysis buffer (Shenggong, Shanghai, China) containing protease inhibitors (Roche) and phosphatase inhibitors (Roche) and briefly sonicated. cells were harvested in RIPA lysis buffer with proteinase K (400 μg/ml) yielding crude cell lysates. Protein concentrations were determined by BCA protein assay (Thermo Fisher scientific). Lysate samples with equivalent protein levels (50 μg) were denatured by incubation at

100 °C for 5 min and electrophoresed on a 10%–15% SDS-polyacrylamide gel. The bands were wet transferred to a nitrocellulose membrane and blocked with 5% non-fat milk. After primary and secondary antibody incubation, images were taken by Syngene and analyzed by ImageJ software. Relative protein expression was normalized to β-actin expression. The relative phosphorylation of target proteins was calculated as the ratios of phosphorylated proteins to target proteins. The related antibody information is summarized in **Table S12**.

Immunofluorescence

For immunofluorescence, KGN cells were seeded onto microscope slides (Millipore, Waltham, Massachusetts, USA). After culture and treatment, cells were fixed with -20℃ pre-cooled acetone. The slides were permeated using 0.5% Triton X-100 in PBS, followed by blocking in 10% normal goat serum. A sufficient amount of diluted primary antibody (CYP19 antibody, 1:100, sc-374176, Santa Cruz Biotechnology, CA, USA; PAI-1 antibody, 1:200, ab222754, Abcam, Cambridge, MA, USA) was added to each slide and incubated in a wet box at 4 ℃ overnight. After blocking with a fluorescent secondary antibody, the slides were then counterstained with DAPI and imaged under a fluorescence microscope (Zeiss).

Aromatase assay

The aromatase activity of KGN or rat GCs was determined using an aromatase (CYP19A) activity assay (ab273306, Abcam) according to the manufacturers' instructions. After cells were subjected to knockdown or over-expression experiments, the cells were incubated with testosterone (10−5 M, Sigma Aldrich) and hMG (500 mIU/mL, Lebaode, Livzon Pharmaceutical

Group Inc, Shanghai, China) for 4 h. Then the cells were collected as cell lysate to conduct aromatase activity assay. Each experiment was performed 3 times independently.

Assay for E1/E2 secretion

Detection of estrone or estradiol secretion is an indirect assay of granulosa cell aromatase activity. KGN or rat GCs were incubated with androstenedione (10−5M, substrate for E1, Selleck, Houston, Texas, USA) or testosterone (10−5 M, substrate for E2, Sigma Aldrich) for 4 h in the presence of hMG (500 mIU/mL, Lebaode, Livzon Pharmaceutical Group Inc, China), and the contents of E1 or E2 secreted into the culture medium were measured by ELISA. For E1 determination, Estrone ELISA kit (KA1908, Abnova, Taipei, China) was used. For E2 determination, Estradiol Parameter Assay Kit (KGE014, R&D) was used.

Phospho-antibody array analysis

The enrichment analysis, including the Kyoto Encyclopedia of Genes and Genomes (KEGG) pathway, biological process, and cellular component, was performed using DAVIA 6.7 (15). Protein interaction networks were built automatically using the Search Tool for the Retrieval of Interacting Genes/Proteins (STRING) system (http://string-db.org/). The Network of protein-protein interaction was built by Cytoscape v2.8.1 (http://www.cytoscape.org). Densely connected regions were calculated by using a graph theoretic clustering algorithm called molecular complex detection (MCODE). Motif enrichment analysis was performed by using MEME (version 4.8.1).

Chomatin Immunoprecipitation (ChIP)

Briefly, 50 μg DNA fragmented to 200–500 bp in size by enzymatic digestion was pre-cleared with Magna ChIP™ Protein A+G Magnetic Beads (Millipore). The pre-cleared DNA was immunoprecipitated with DNMT3A (CST, 49768S). Immunoglobin G (IgG) was used as the negative control. ChIP products were collected using MinElute PCR Purification Kit (Qiagen). The qPCR results were calculated as IP/1% input as follows: (IP − IgG) / (Input − IgG).

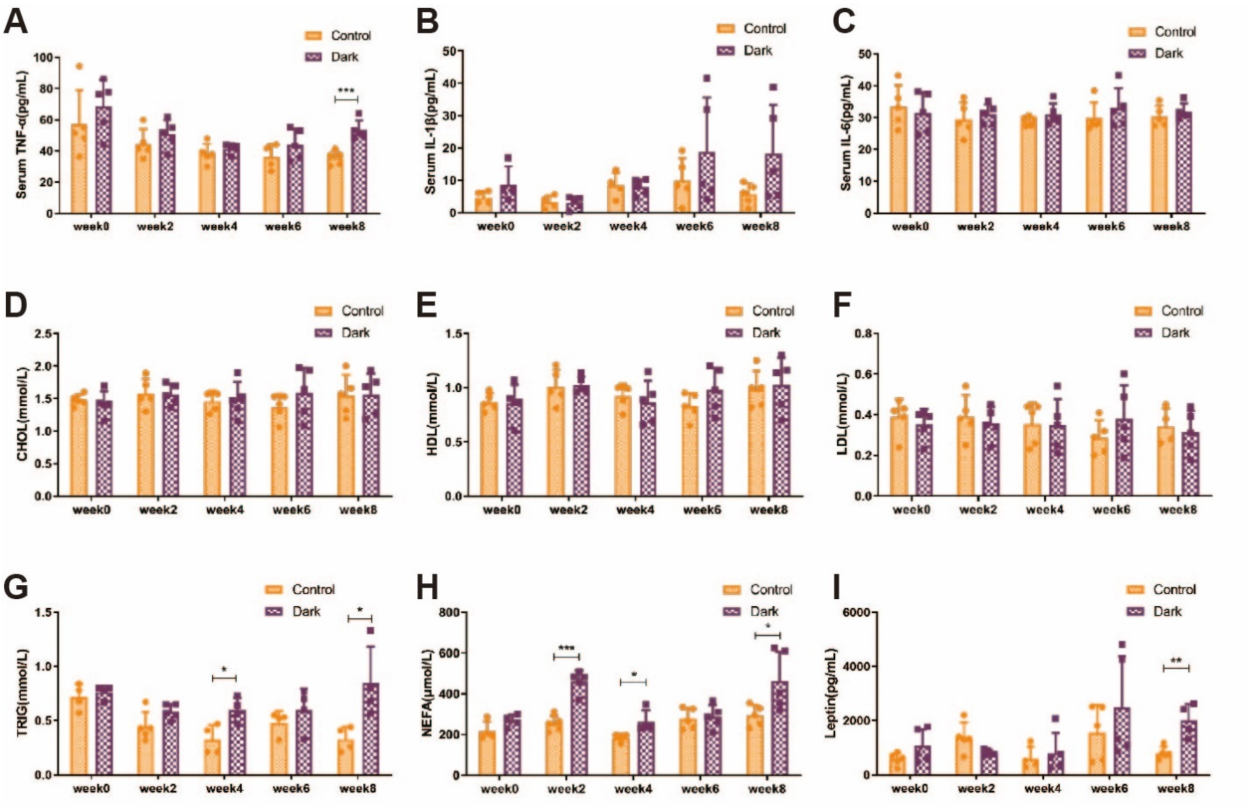

Figure S1.

Continuous dark exposure leads to multiple alterations in inflammatory and metabolic parameters.

(A–I) TNF-α concentration (A), IL-1β concentration (B), and IL-6 concentration (C) in rat serum are measured using ELISA (n = 4–5). TNF-α concentration (A), IL-1β concentration (B), and IL-6 concentration (C) in rat serum are measured using ELISA (n = 4–5). CHOL concentration (D), HDL-C concentration (E), LDL-C concentration (F), TRIG concentration (G), NEFA concentration (H) and leptin concentration (I) in rat serum are measured using an autoanalyzer (n = 4–5). TNF-α, tumor necrosis factor-alpha; IL-1β, interleukin 1 beta; IL-6, interleukin 6; CHOL, cholesterol; HDL, high-density lipoprotein cholesterol; LDL, low-density lipoprotein cholesterol; TRIG, triglycerides; NEFA, nonesterified fatty acid. Data are shown as mean ± SD. *$P < 0.05$, **$P < 0.01$, ***$P < 0.001$ (Student's *t* test).

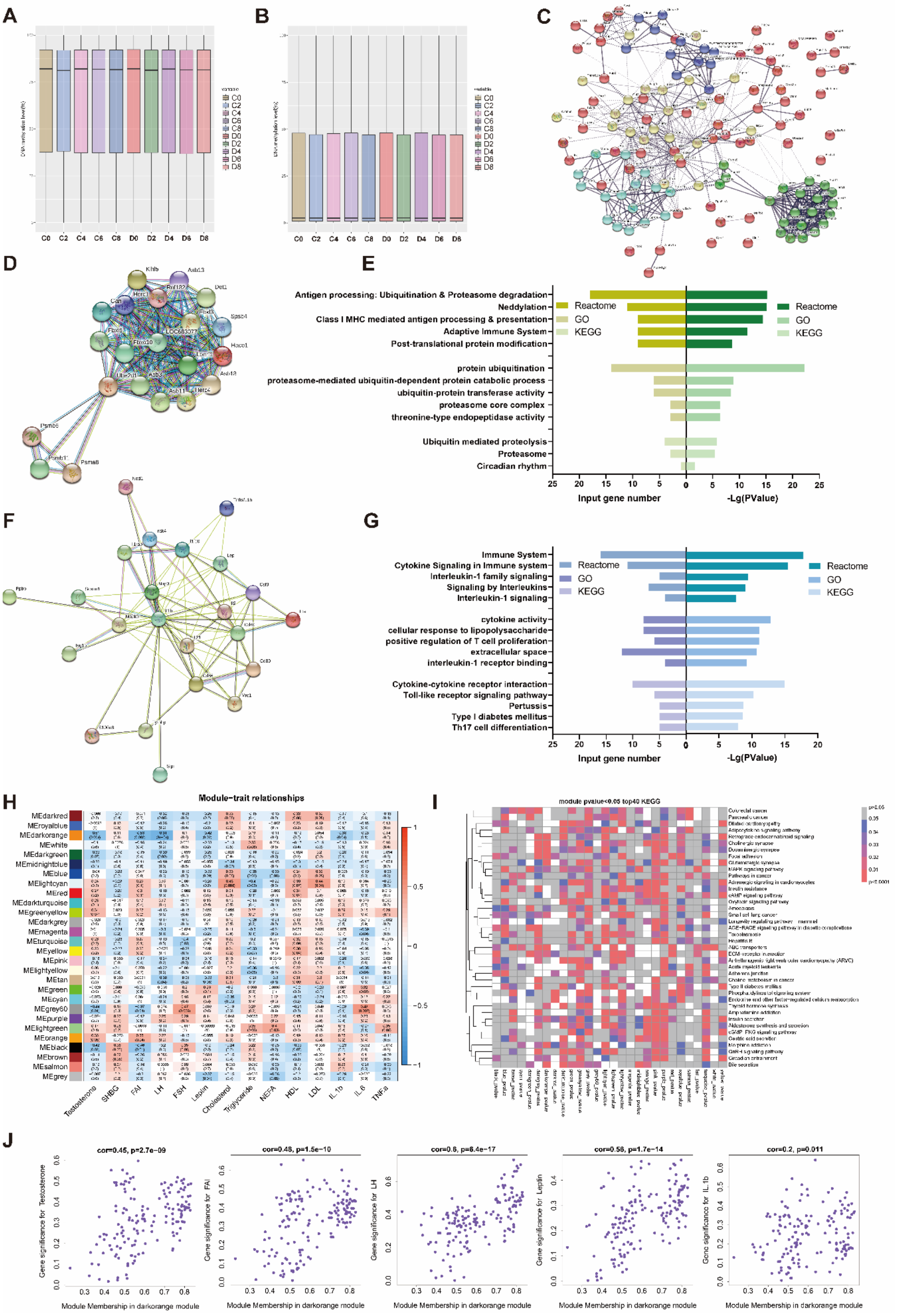
A
B
C
D
E
F
G
H
I
J
C0
C2
C4
C6
C8
D0
D2
D4
D6
D8
Khlb
Asb13
Rnf182
Dcf1
Herc1
Fbxl3
Spsb4
Fbxl5
LOC683077
Haca1
Fbxo10
Ube2d1
Asb3
Asb11
Herc4
Asb18
Psmb6
Psmb11
Psma8
Antigen processing: Ubiquitination & Proteasome degradation
Neddylation
Class I MHC mediated antigen processing & presentation
Adaptive Immune System
Post-translational protein modification
protein ubiquitination
proteasome-mediated ubiquitin-dependent protein catabolic process
ubiquitin-protein transferase activity
proteasome core complex
threonine-type endopeptidase activity
Ubiquitin mediated proteolysis
Proteasome
Circadian rhythm
Reactome
GO
KEGG
Input gene number
-Lg(PValue)
Immune System
Cytokine Signaling in Immune system
Interleukin-1 family signaling
Signaling by Interleukins
Interleukin-1 signaling
cytokine activity
cellular response to lipopolysaccharide
positive regulation of T cell proliferation
extracellular space
interleukin-1 receptor binding
Cytokine-cytokine receptor interaction
Toll-like receptor signaling pathway
Pertussis
Type I diabetes mellitus
Th17 cell differentiation
Module-trait relationships
MEdarkred
MEroyalblue
MEdarkorange
MEwhite
MEdarkgreen
MEmidnightblue
MEblue
MElightcyan
MEred
MEdarkturquoise
MEgreenyellow
MEdarkgrey
MEmagenta
MEturquoise
MEyellow
MEpink
MElightyellow
MEtan
MEgreen
MEcyan
MEgrey60
MEpurple
MElightgreen
MEorange
MEblack
MEbrown
MEsalmon
MEgrey
Testosterone
SHBG
FAI
LH
FSH
Leptin
Cholesterol
Triglyceride
NEFA
HDL
LDL
IL-1b
IL-6
TNFa
module pvalue<0.05 top40 KEGG
cor=0.45, p=2.7e-09
cor=0.48, p=1.5e-10
cor=0.6, p=6.4e-17
cor=0.56, p=1.7e-14
cor=0.2, p=0.011
Gene significance for Testosterone
Gene significance for FAI
Gene significance for LH
Gene significance for Leptin
Gene significance for IL-1b
Module Membership in darkorange module

Figure S2.

Changes caused by darkness are enriched in neural, metabolic and inflammatory pathways

(A) Boxplot showing the overall distribution of the methylation of CpG loci in gene body regions in each rat ovarian granulosa cell sample. The short bold bar in the box represents average methylation. Sample numbers are stated in the bottom of each box. *P* values are calculated using a two-tailed Student's *t* test.

(B) Boxplot showing the overall distribution of the methylation of CpG loci in promoter regions in each rat ovarian granulosa cell sample. The short bold bar in the box represents average methylation. Sample numbers are stated in the bottom of each box. *P* values are calculated using a two-tailed Student's *t* test.

(C) STRING protein network analysis of the 1608 genes in first cluster (red) of STEM analysis (Figure 2C), which is divided into 5 clusters according to K-Means clustering algorithm.

(D) STRING protein network analysis of the 21 genes in green cluster of STRING analysis (Figure S2C).

(E) Functional annotation charts using Reactome/GO/KEGG performed on the 21 genes in green cluster of STRING analysis (Figure S2C). Significance is indicated as −log10 *P* value.

(F) STRING protein network analysis of the 23 genes in yellow cluster of STRING analysis (Figure S2C).

(G) Functional annotation charts using Reactome/GO/KEGG performed on the23 genes in yellow cluster of STRING analysis (Figure S2C). Significance is indicated as −log10 *P* value.

(H) Correlation analysis between phenotypic indicators and WGCNA modules through WGCNA co-methylation network and module-trait correlation analysis (also see method). The total 28 modules are labeled with different colors. The columns correspond to phenotypic indicators. The

color of each cell indicates the correlation coefficient between the module and phenotypic indicator (the top number in the cell represents the correlation coefficient, and the bottom one in parentheses represents the *P* value).

(I) Correlation analysis between KEGG pathways and WGCNA modules. Heatmap color indicates the *P* value per functional category. The color scale of the *P* value is shown on the right.

(J) Scatter plots of the MM in the darkorange module vs GSs for different phenotypic indicators. Significantly high correlations between MM in the darkorange module and Testosterone / FAI / LH / Leptin / IL-1β are found. The correlation and the *P* value are shown above the plots.

WGCNA, Weighted gene expression network analysis; GS, gene significance; MM, module membership; FAI, free androgen index; LH, luteinizing hormone; IL-1β, interleukin 1 beta.

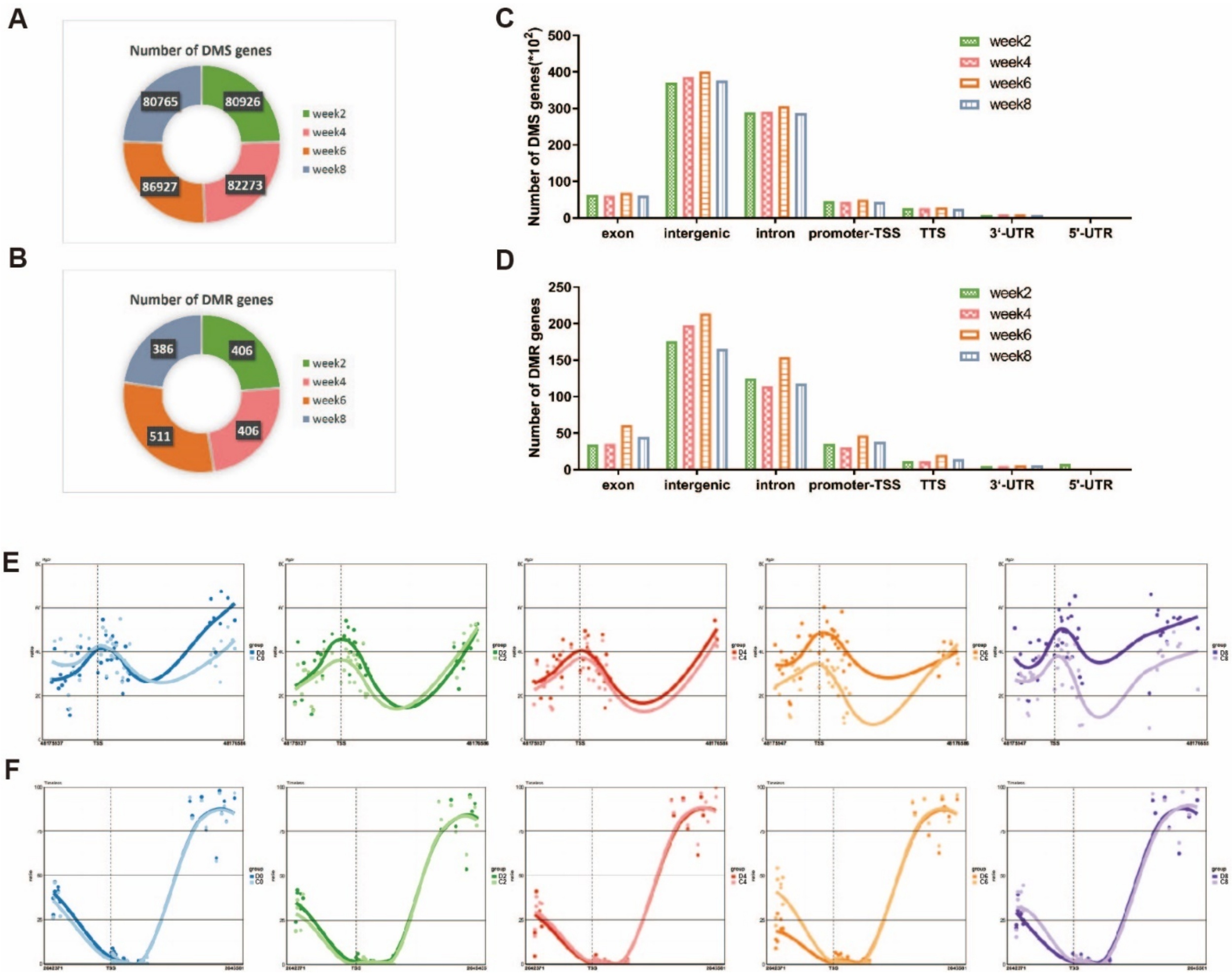

Figure S3.

Differential methylation at different time points and in different gene elements

(A) Pie graph of the numbers of DMS genes at different time points.

(B) Pie graph of the numbers of DMR genes at different time points.

(C) The number of DMS genes in exons, intergenic regions, introns, promoter TSSs, TTSs, 3'UTR or 5'UTR is shown for different time points.

(D) The number of DMR genes in exons, intergenic regions, introns, promoter TSSs, TTSs, 3'UTR or 5'UTR is shown for different time points.

(E) A schematic representation of the changes in CpG sites in the promoter region of Igf2r at week 0, 2, 4, 6, or 8, respectively.

(F) A schematic representation of the changes in CpG sites in the promoter region of Timeless at week 0, 2, 4, 6, or 8, respectively.

DMS, differentially methylated sites; DMR, differentially methylated regions; TSS, transcription start sites; TTS, transcription termination sites; UTR, Untranslated region.

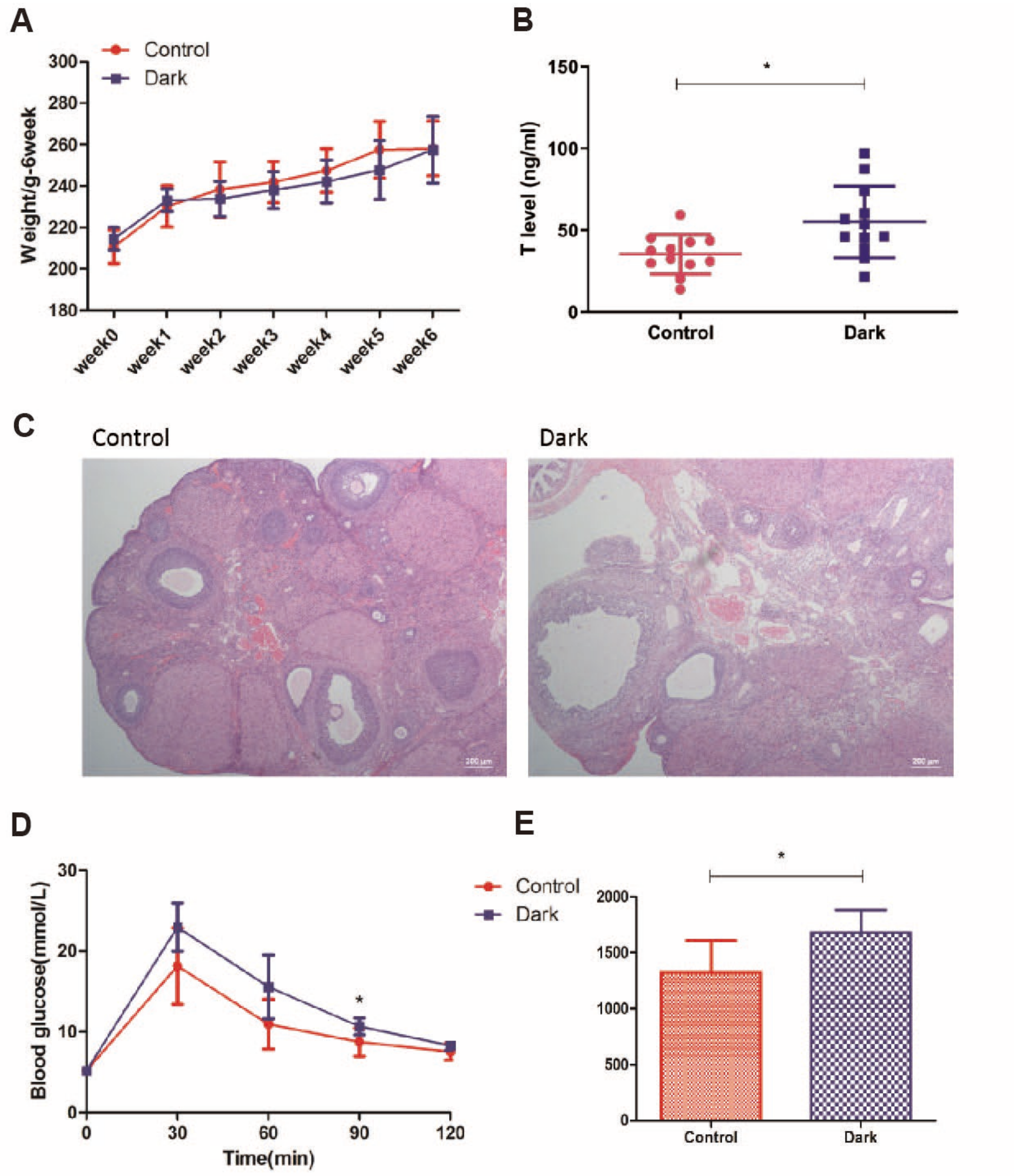

Figure S4.

PCOS-like phenotypes and abnormal glucose metabolism of rats in continuous darkness for 6 weeks

(A) Body mass of dark rats and control rats at week 6 (n = 8).

(B) Testosterone concentrations in the serums of dark rats and control rats are measured using ELISA (n = 12).

(C) Representative images of H&E staining of ovaries from controls and dark group, scale bars, 200 μm.

(D–E) The IPGTT of rats (D) and the corresponding glucose area under the curve (AUC, E) during the IPGTT (control group: n = 7; dark group: n = 8).

Data are shown as mean ± SD. *$P < 0.05$ (Student's *t* test).

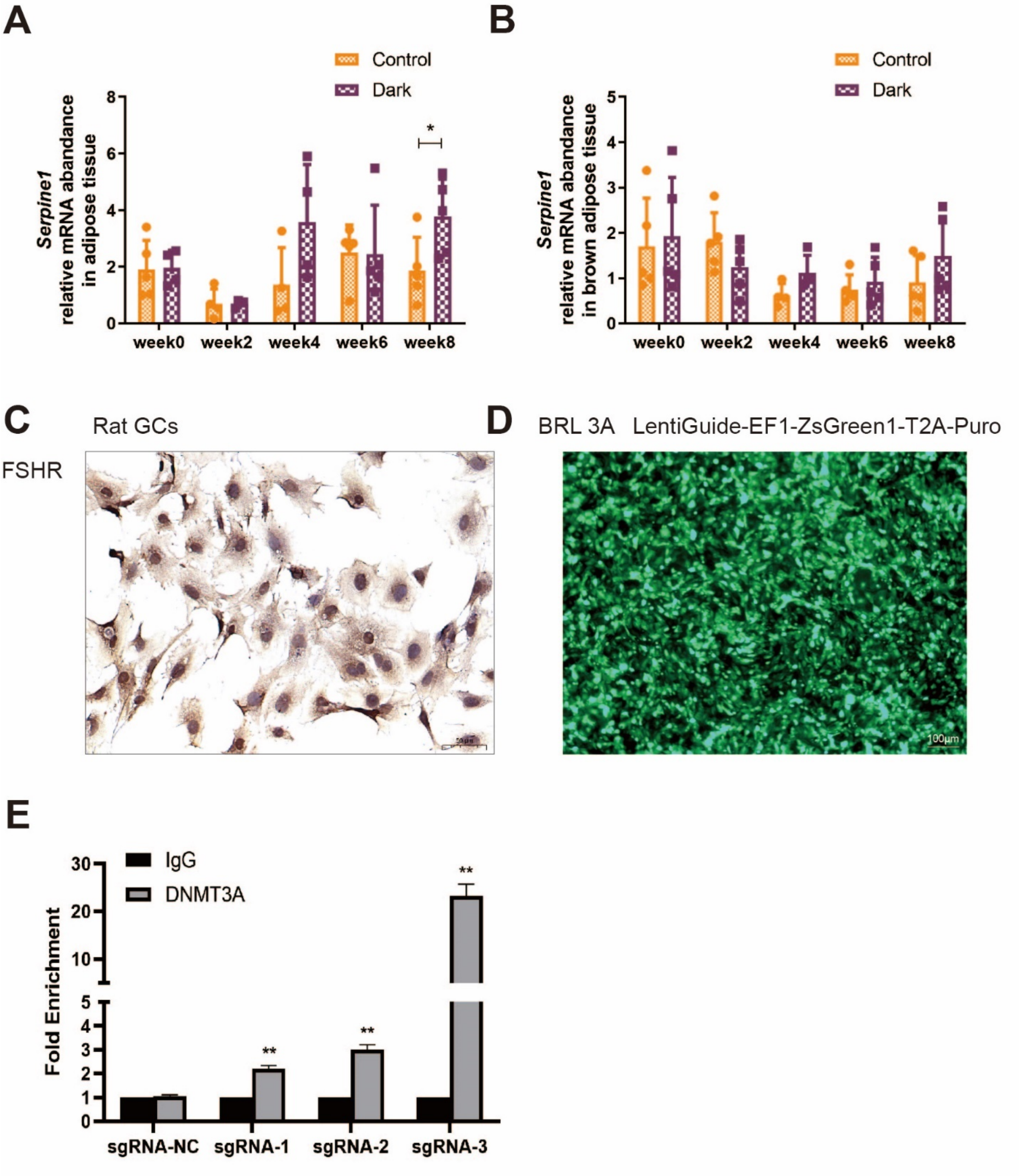

Figure S5.

Serpine1 expressions in adipose tissue and brown adipose tissue. DNMT3A binds to the target Serpine1 promoter region successfully.

(A) Quantitative real-time PCR analysis for the dynamic expression of Serpine1 in rat adipose tissue during 8 weeks (n = 4–5).

(B) Quantitative real-time PCR analysis for the dynamic expression of Serpine1 in rat brown adipose tissue during 8 weeks (n = 4–5).

(C) Identification of primary rat ovarian granulosa cells by FSHR immunohistochemistry, scale bars, 50 μm.

(D) Identification of the successful transfection of the sgRNA-ZsGreen1-T2A-Puro system by the green fluorescence protein (ZsGreen1) in BRL 3A cells, scale bars, 100 μm.

(E) ChIP-PCR performed with antibodies to DNMT3A or nonspecific IgG antibody control in BRL 3A cells infected with GPLVX-CMV-3×Flag-NLS-dCas9-Dnmt3aCD-T2A-Blasticidin vectors and sgRNA-ZsGreen1-T2A-Puro combined with sgRNA-1, 2, 3 or sgRNA-NC. sgRNA-NC was used as a negative control.

ChIP, Chromatin immuno-precipitation. Data are shown as mean ± SD. $*P < 0.05$, $**P < 0.01$ (Student's *t* test).

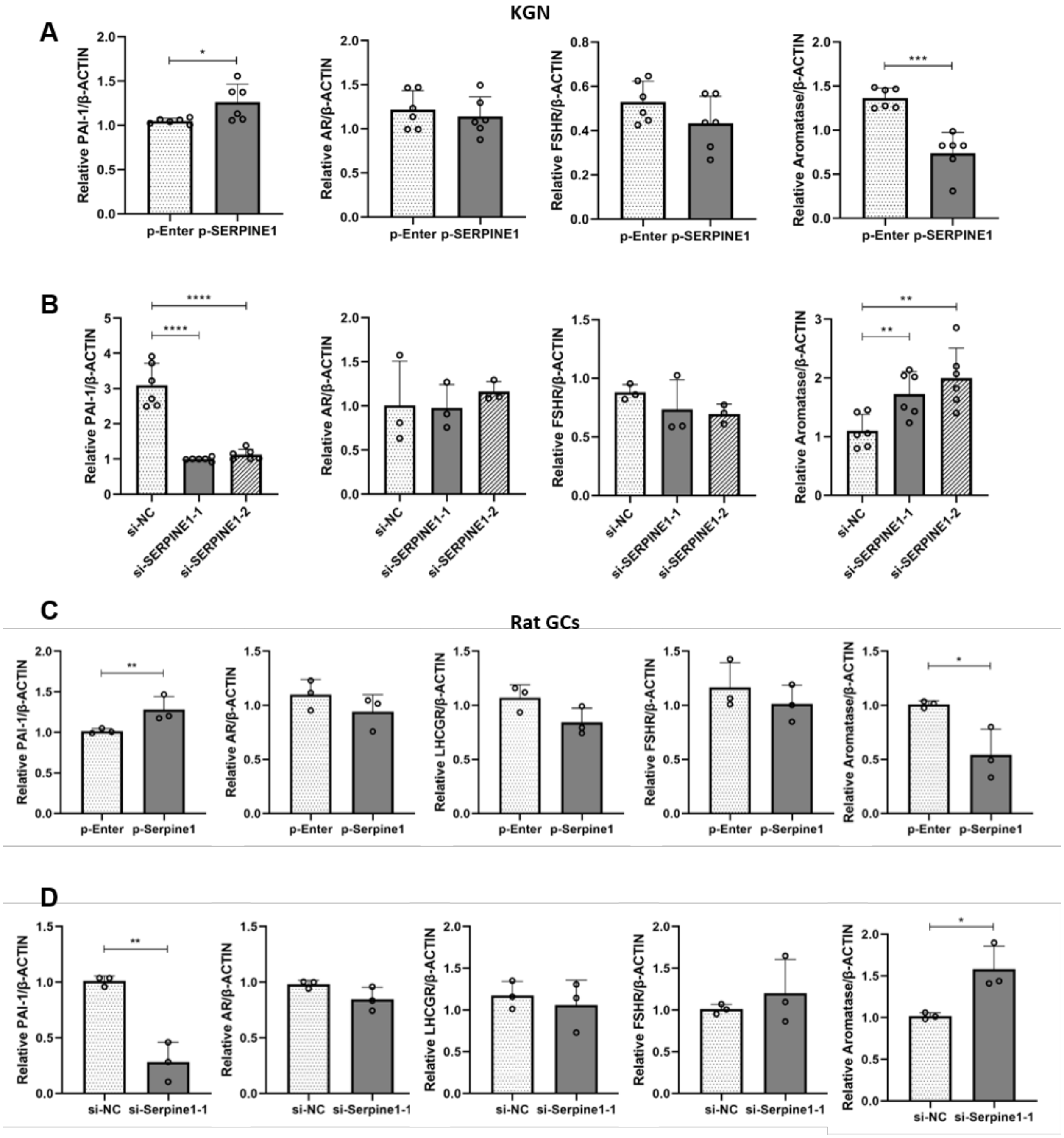

Figure S6.

The quantification of protein expression in Figure 4.

(A) The quantification of protein expression of Figure 4E.

(B) The quantification of protein expression of Figure 4G.

(C) The quantification of protein expression of Figure 4I.

(D) The quantification of protein expression of Figure 4K.

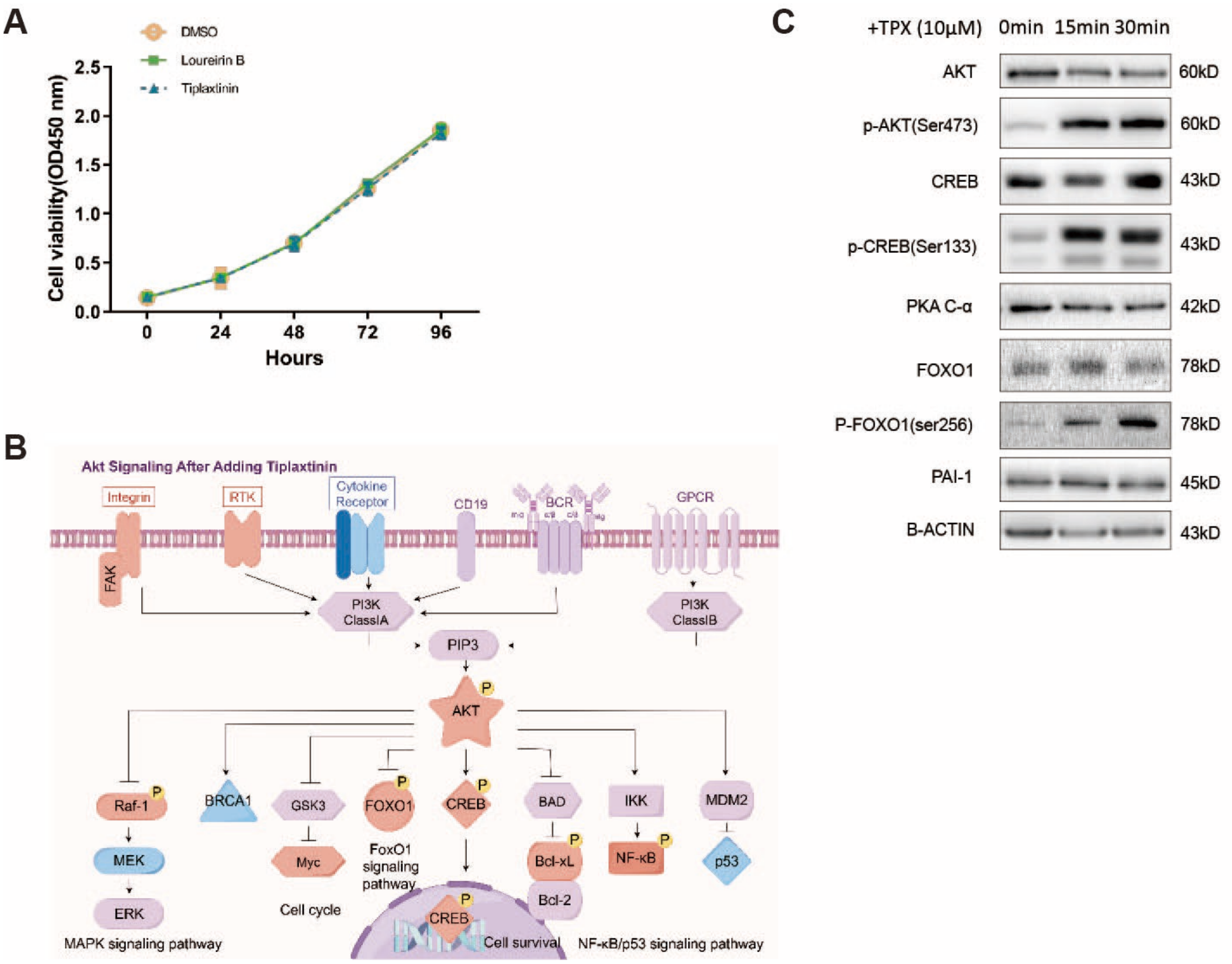

Figure S7.

Inhibition of SERPINE1 promotes AKT phosphorylation.

(A) Cell growth curves after SERPINE1 inhibitors LrB and TPX treatments in KGN cells using the Cell Counting Kit-8 assay.

(B) Schematic illustration of phosphorylation changes in the AKT pathway after addition of TPX treatment (10 μM) for 15 min. Proteins upregulated at the phosphorylation level are shown in red and proteins downregulated at the phosphorylation level are shown in blue.

(C) Western blot analysis of expression levels of proteins related to AKT signaling pathway, CREB signaling pathway, FOXO1 signaling pathway, and PAI-1 at 15 min and 30 min after the addition of TPX (10 μM) in KGN cells.

Data are shown as mean ± SD. One-way ANOVA.

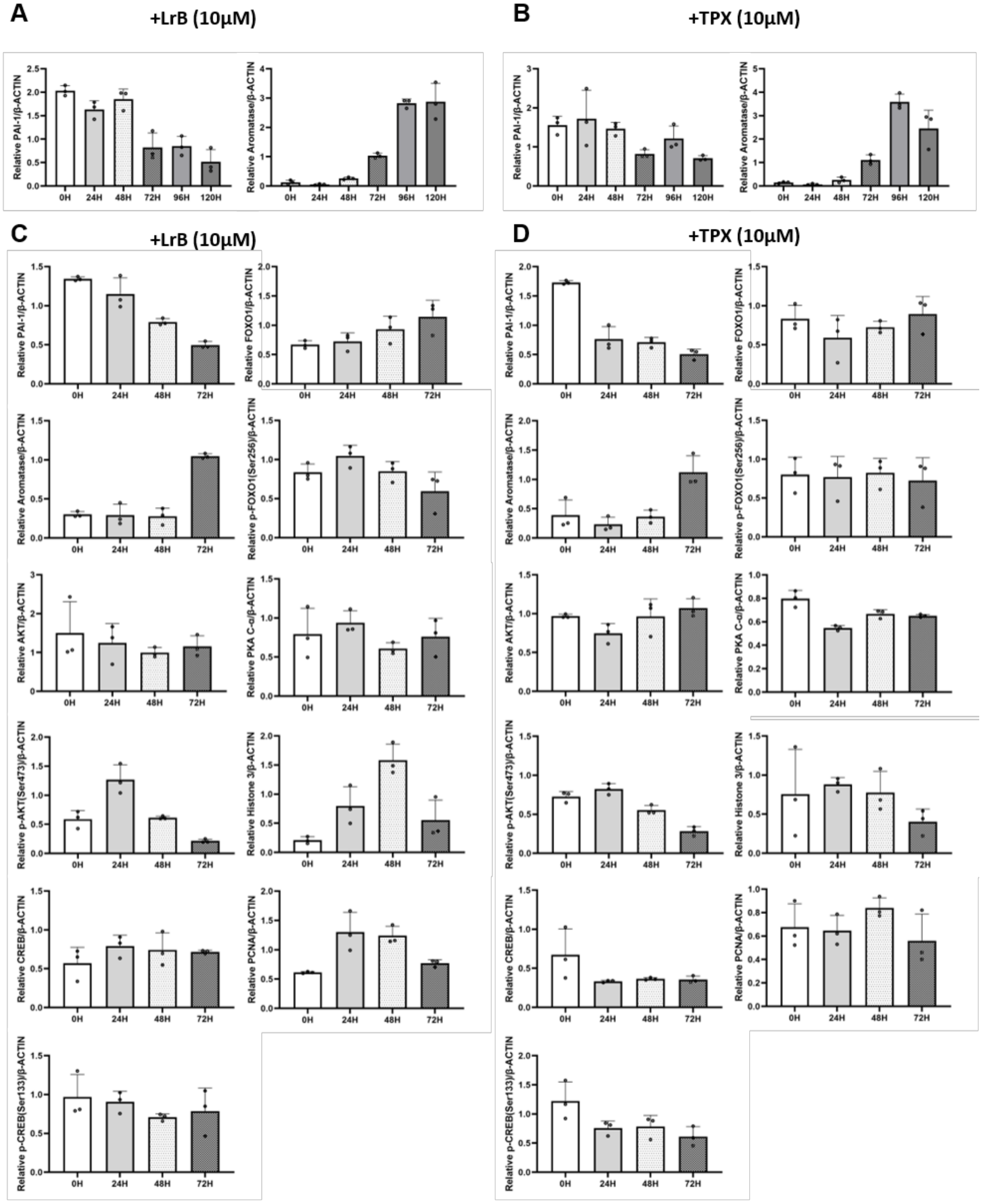

Figure S8.

The quantification of protein expression in Figure 5.

(E) The quantification of protein expression of Figure 5C.

(F) The quantification of protein expression of Figure 5D.

(G) The quantification of protein expression of Figure 5J.

(H) The quantification of protein expression of Figure 5K.

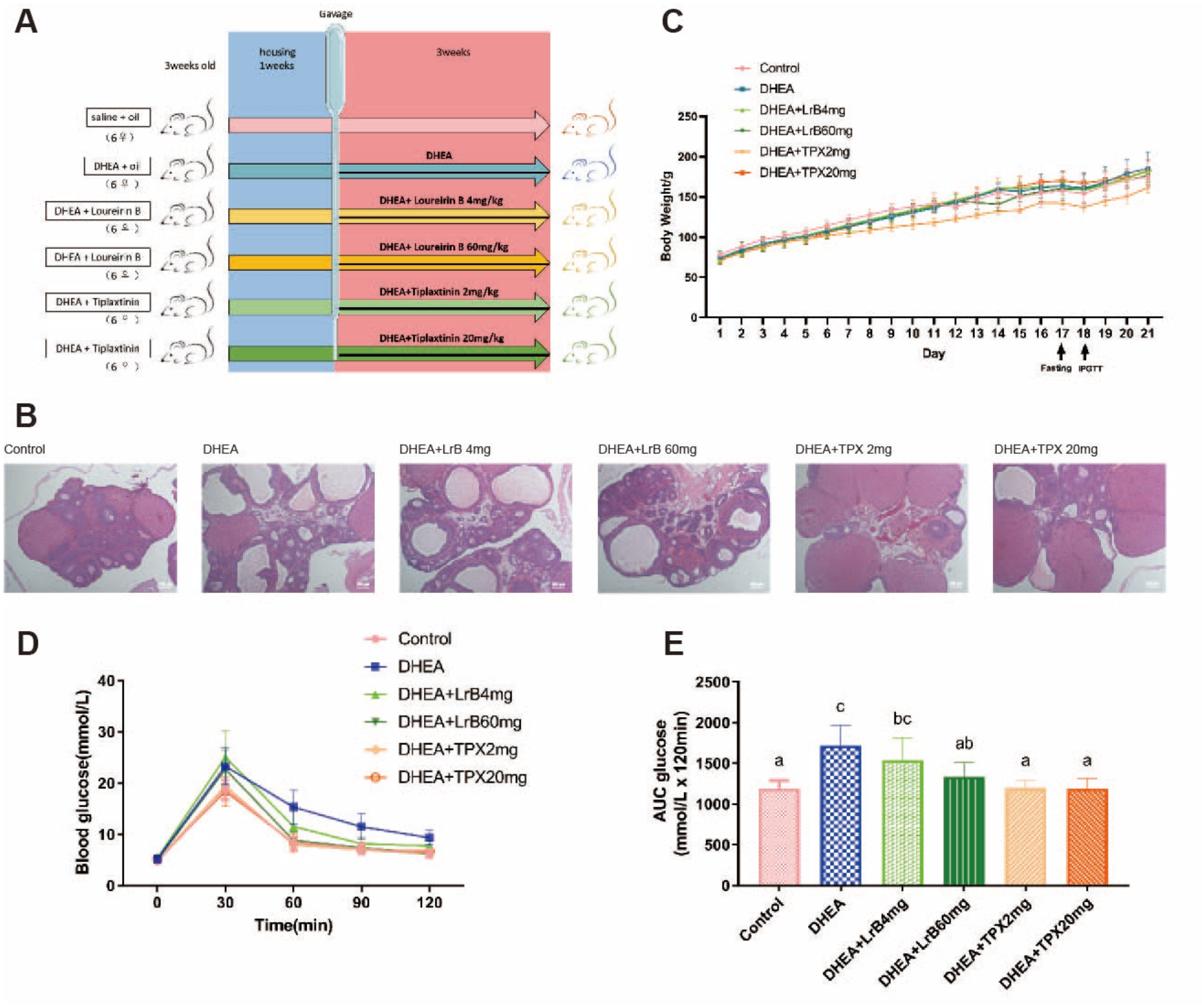

Figure S9.

Pre-experiment on the effect of the addition of different concentrations of PAI-1 inhibitor to the phenotypes of DHEA-treated rats.

(A) Schematic illustration of DHEA modelling and different concentration of Loureirin B (4 mg/kg or 60 mg/kg) or Tiplaxtinin (2 mg/kg or 20 mg/kg) treated modelling design.

(B) Representative images of H&E staining of ovaries from controls (saline + oil), DHEA group (DHEA + oil), DHEA combined Loureirin B treatment group (DHEA + LrB 4 mg/kg), DHEA combined Loureirin B treatment group (DHEA + LrB 60 mg/kg), DHEA combined Tiplaxtinin

treatment group (DHEA + TPX 2 mg/kg), and DHEA combined Tiplaxtinin treatment group (DHEA + TPX 20 mg/kg), scale bars, 200 μm.

(C) Body weight curves of DHEA modelling and different concentration of Loureirin B (4 mg/kg or 60 mg/kg) or Tiplaxtinin (2 mg/kg or 20 mg/kg) treated modelling rats during 21 days.

(D–E) The IPGTT of rats in 6 groups (D) and the corresponding glucose area under the curve (AUC, E) during the IPGTT. Different letters (a, b, c) in the same column indicate significant differences ($P < 0.05$, one-way ANOVA)

Data are shown as mean ± SD. One-way ANOVA.

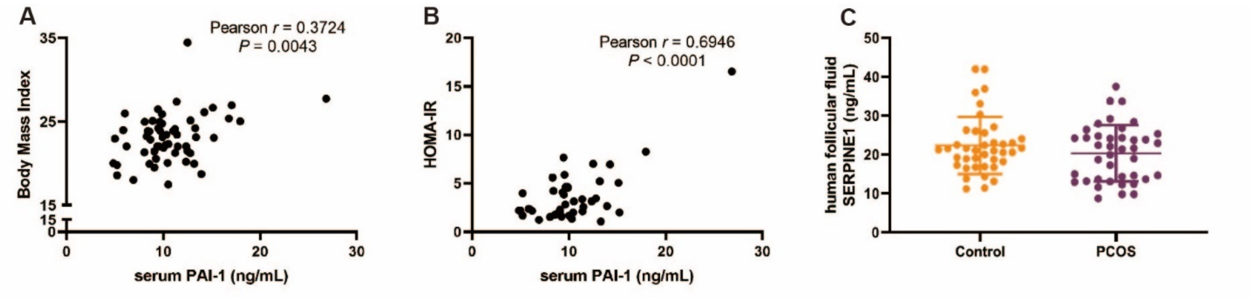

Figure S10.

SERPINE1 is upregulated and hypomethylated in PCOS patients

(A) Pearson analysis between BMI and PAI-1 levels from 57 patients with PCOS (n = 57, Pearson *r* = 0.3724, *P* = 0.0043).

(B) Pearson analysis between HOMA-IR and PAI-1 levels from 57 patients with PCOS (n = 57, Pearson *r* = 0.6946, *P* < 0.0001).

(C) ELISA array of PAI-1 levels in follicular fluid from a cohort including 40 PCOS patients and 40 controls (*P* = 0.2183).

BMI, Body Mass Index; HOMA-IR, Homeostatic Model Assessment of Insulin Resistance. Data are shown as mean ± SD. Pearson *r* test in (A), (B), Student's *t* test in (C). **P*<0.05, ***P*<0.01, ****P*<0.001.

Table S1.

Clinical and biochemical data of women with and without PCOS involved in serum ELISA

	PCOS (n=57)	Control (n=57)	P-value
Age, years	31.64±2.91	31.55±3.49	0.8785
BMI, kg/m2	22.94±2.80	22.46±1.55	0.3460
LH, IU/L	12.05±6.94	4.66±1.38	＜0.0001
FSH, IU/L	5.79±1.13	6.67±1.90	0.0226
E2, pg/mL	48.19±22.04	34.69±15.03	＜0.0001
Prolactin, ng/mL	20.89±17.84	16.79±8.25	0.2500
T, ng/dL	48.53±15.74	23.09±8.28	＜0.0001
LH/FSH	2.14±1.29	0.72±0.22	＜0.0001
AMH, ng/ml	10.02±4.60	2.81±3.37	＜0.0001
GLU, mmol/L	5.60±1.69	5.25±0.62	0.1116
FINS, mIU/L	14.42±7.76	N/A	N/A
HOMA-IR	3.64±2.85	N/A	N/A
TSH, uIU/mL	2.83±2.24	2.58±1.47	0.8608
TC, mmol/L	4.53±0.81	4.21±0.95	0.0649
TG, mmol/L	1.30±0.83	0.89±0.87	0.0165
HDL-C, mmol/L	1.35±0.38	1.45±0.33	0.0005
LDL-C, mmol/L	2.95±0.61	2.51±0.61	0.0005

Data are presented as mean ± SD. Student t-test was used in the analysis of the data (age, TC, TG, HLDL-C, LDL-C) with normal distribution. Mann-Whitney test was used to compare differences between patients with and without PCOS in non-normal distribution parameters.

PCOS, polycystic ovary syndrome; BMI, body mass index; LH, luteinizing hormone; FSH, follicle-stimulating hormone; E2, estradiol; T, testosterone; AMH, Antimüllerian hormone; GLU, fasting blood glucose; FINS, fasting blood insulin; HOMA-IR, homeostasis model assessment-insulin resistance; TSH, thyroid-stimulating hormone; TC, total cholesterol; TG, triglyceride*; HDL-C, high-density lipoprotein cholesterol; LDL-C, low-density lipoprotein cholesterol.*

Table S2.

Clinical and biochemical data of women with and without PCOS involved in follicular fluid ELISA

	PCOS (n=40)	Control (n=40)	P-value
Age, years	29.48±3.44	29.10±3.24	0.6173
BMI, kg/m2	25.41±4.06	22.52±2.87	0.0010
LH, IU/L	9.71±6.67	5.15±1.95	＜0.0001
FSH, IU/L	5.82±1.05	6.59±1.43	0.0103
E2, pg/mL	37.37±17.91	42.04±18.68	0.2603
Prolactin, ng/mL	16.16±6.57	16.87±7.03	0.6388
T, ng/dL	38.92±15.67	25.78±12.48	＜0.0001
LH/FSH	1.65±1.05	0.81±0.31	＜0.0001
AMH, ng/ml	8.54±4.12	3.94±2.37	＜0.0001
GLU, mmol/L	5.56±0.84	5.14±0.40	0.0033
FINS, mIU/L	21.12±11.44	N/A	N/A
HOMA-IR	5.57±3.48	N/A	N/A
TSH, uIU/mL	2.37±1.02	2.29±0.88	0.6858
TC, mmol/L	4.29±1.00	4.09±0.59	0.6492
TG, mmol/L	1.51±1.89	0.92±0.33	0.0677
HDL-C, mmol/L	1.25±0.25	1.51±0.33	0.0010
LDL-C, mmol/L	2.64±0.81	2.44±0.52	0.2672

Data are presented as mean ± SD. Student t-test was used in the analysis of the data (age, FSH, E2, PRL, T, TSH, TC) with normal distribution. Mann-Whitney test was used to compare differences between patients with and without PCOS in non-normal distribution parameters.

PCOS, polycystic ovary syndrome; BMI, body mass index; LH, luteinizing hormone; FSH, follicle-stimulating hormone; E2, estradiol; T, testosterone; AMH, Antimüllerian hormone; GLU, fasting blood glucose; FINS, fasting blood insulin; HOMA-IR, homeostasis model assessment-insulin resistance; TSH, thyroid-stimulating hormone; TC, total cholesterol; TG, triglyceride*; HDL-C, high-density lipoprotein cholesterol; LDL-C, low-density lipoprotein cholesterol.*

Table S3.

Clinical and biochemical data of women with and without PCOS involved in pyrosequencing

	PCOS (n=10)	Control (n=10)	P-value
Age, years	31.60±3.78	31.00±2.45	0.6833
BMI, kg/m2	22.28±3.02	22.27±1.12	0.9925
LH, IU/L	15.09±4.84	4.91±1.26	＜0.0001
FSH, IU/L	5.44±1.48	7.33±1.76	0.0214
E2, pg/mL	56.63±12.54	33.98±11.72	0.0007
Prolactin, ng/mL	21.76±16.86	14.91±4.52	0.2322
T, ng/dL	58.26±11.07	22.13±9.26	＜0.0001
LH/FSH	2.97±1.21	0.68±0.16	＜0.0001
AMH, ng/ml	13.12±3.08	2.20±0.93	＜0.0045
GLU, mmol/L	6.39±3.69	5.15±0.22	0.3310
FINS, mIU/L	15.69±8.40	N/A	N/A
HOMA-IR	4.80±4.55	N/A	N/A
TSH, uIU/mL	2.22±1.54	2.63±1.32	0.5317
TC, mmol/L	5.05±1.25	4.57±0.64	0.3276
TG, mmol/L	1.58±1.38	0.81±0.31	0.1199
HDL-C, mmol/L	1.38±0.47	2.89±0.67	0.5593
LDL-C, mmol/L	3.19±0.73	1.49±0.32	0.3813

Data are presented as mean ± SD. Student t-test was used in the analysis of the data (age, FSH, E2, PRL, T, TSH, TC) with normal distribution. Mann-Whitney test was used to compare differences between patients with and without PCOS in non-normal distribution parameters.

PCOS, polycystic ovary syndrome; BMI, body mass index; LH, luteinizing hormone; FSH, follicle-stimulating hormone; E2, estradiol; T, testosterone; AMH, Antimüllerian hormone; GLU, fasting blood glucose; FINS, fasting blood insulin; HOMA-IR, homeostasis model assessment-insulin resistance; TSH, thyroid-stimulating hormone; TC, total cholesterol; TG, triglyceride*; HDL-C, high-density lipoprotein cholesterol; LDL-C, low-density lipoprotein cholesterol.*

Table S4.

sgRNA oligo sequences

oligo	5'to 3'
Primer-NC-T	caccgACGGAGGCTAAGCGTCGCAA
Primer-NC-B	aaacTTGCGACGCTTAGCCTCCGTc
Primer-T1	CACCGCACGTGTCCCAGCAAGTTAC
Primer-B1	AAACGTAACTTGCTGGGACACGTGC
Primer-T2	CACCGCCTTTATACCAGATGTGGGC
Primer-B2	AAACGCCCACATCTGGTATAAAGGC
Primer-T3	CACCGGCTCCCTTTATACCAGATG
Primer-B3	AAACCATCTGGTATAAAGGGAGCC

Table S5.

Primers for the pyrosequencing analysis of the rat *Serpine1* promoter region-set1

Primer Set 1		
**Primer**	**Id**	**Sequence**
PCR	F1	GTATAGTTGGATTAGGTTGTTGTAGA
PCR	R1	CCTCTAAAAAAAAATATTTCCTACTTTCAT
Sequencing	S1	ATTAGGTTGTTGTAGATTTT
Target Polymorphisms	Position1, Position2, Position3, Position4, Position5, Position6	
Sequence to Analyze	YGAGAGTTTT GTGAAGGAGG AAYGTTGTAT ATTYGTTTTT YGTAGTATAT AGTTAATTAT AGTTGAGYGA TAGTTAATAA GAGTTAATTA TAAGGTATTT TYGAAAGTTT TAGGTAGGAG AAAAGTAA	
Amplicon length	218	

Table S6.

Primers for the pyrosequencing analysis of the rat *Serpine1* promoter region-set2

**Primer Set 1**		
**Primer**	**Id**	**Sequence**
PCR	F2	GTTAAAGGTAAAGGGATAGAGATGTT
PCR	R2	CCACCCACCTTCTAACTCTAAA
Sequencing	S2	ATTTGTTTAATTATTTTTTATGTTT
Target Polymorphisms	Position7, Position8, Position9, Position10	
Sequence to Analyze	TTTTATATAY GTATATATAY GTGTTTTAGT AAGTTATTGG GAGGGAGGGA AGGAGAGAGG GGGTAGGGYG GGYGAGTAGT TTAGATATTT TTAGAGTTA	
Amplicon length	180	

Table S7.

Primers for the pyrosequencing analysis of the rat *Line-1* gene

**Primer Set 1**		
**Primer**	**Id**	**Sequence**
PCR	F	GYGGTATAGGTTTTTTTGGTTGTT
PCR	BR	AATTCACCAAACAACTTTCTTACAA
Sequencing	FS	TAGGTTTTTTTGGTTGTTG
Sequence to Analyze	TYGTTGTA	

Table S8.

Primers for the pyrosequencing analysis of the human *SERPINE1* gene promoter region

**Primer Set 1**		
**Primer**	**Id**	**Sequence**
PCR	1F	ATAAGAGAGTTTTTAGGGGTATAGAGA
⬐ PCR	1BR	CCAACAACCACAAAACATACAAC
→ Sequencing	1FS1	AGGGGTATAGAGAGAGTTTGGATA
Sequence to Analyze	**CG**TGGGGAGTCAGC**CG**TGTATCAT**CG**GAGG**CG**GC**CG**GGCACA	
→ Sequencing	1FS2	TAGATAGATAAAATTTAGATAATTA
Sequence to Analyze	**CG**TGGCTGGCTGCA	
**Primer Set 2**		
**Primer**	**Id**	**Sequence**
PCR	2F	TGGTTGGTTGTATGTTTTGTGG
⬐ PCR	2BR	AAATTATCAAAAATAACCTCCATCA
→ Sequencing	2FS1	TGGTTGTTGGGTTGGGTTTAG
Sequence to Analyze	GAGGAGGGAGGGG**CG**	
→ Sequencing	2FS2	TTTTTTTGGAGGTGGTTTAGAGTAT

Sequence to Analyze	CGGGTGGACAGCCCTGGGGGAAAACTTCCACGTTTTGA	
**Primer Set 3**		
**Primer**	**Id**	**Sequence**
PCR	3F	TTTTGATGGAGGTTATTTTTGATAA
← PCR	3BR	CCTTAACCTTTCTACCCTCTACCTA
→ Sequencing	3FS1	TTGATAATTTTATAGTGATTTGGTT
Sequence to Analyze	CGCCAAAGGAAAAGCAGGCAACGTGAG	
→ Sequencing	3FS2	GGTTTTAGGTTTTTTGGGTTATT
Sequence to Analyze	CGGCATGGCAGACAGTCAACCTGGCAGGACATCCGGGAGA	

Table S9.

Primer sequences of genes mentioned in the article of qPCR(Human)

Target genes	Primer sequences(5’ → 3’)	Amplification condition
ACTB	F:5’-CTCCATCCTGGCCTCGCTGT-3’	Stage 1: 95°C, 10s<br>Stage 2: 95°C, 10s<br>60°C, 20s<br>72°C, 20s<br>Number of cycles: 40<br>Stage 3: 95°C, 5s<br>65°C, 1min<br>4°C, ∞
	R:5’-GCTGTCACCTTCACCGTTCC-3’	
DNMT1	F:5’-GTGGGGGACTGTGTCTCTGT-3’	
	R:5’-TGAAAGCTGCATGTCCTCAC-3’	
SERPINE1	F:5’-CTCTCTCTGCCCTCACCAAC-3’	
	R:5’-GTGGAGAGGCTCTTGGTCTG-3’	
AGT	F:5’-GAACTGGATGTTGCTGCTGA-3’	
	R:5’-GGAGAAGCCCTTCATCTTCC-3’	
CYP19A1	F:5’-CCAGTGAAAAAGGGGACAAA-3’	
	R:5’-CCATGGCGATGTACTTTCCT-3’	
SRD5A1	F:5’-ACCAAGGGGAGGCTTATTTG-3’	
	R:5’-TTTCCGGAGGTACCACTCAT-3’	
CYP11A1	F:5’-GGAAATTACTCGGGGGACAT-3’	
	R:5’-CACATGGTCCTTCCAGGTCT-3’	
HSD3B1	F:5’-AGAGGCCTGTGTCCAAGCTA-3’	
	R:5’-TTTTGCTGTGTGGGTATGGA-3’	
STAR	F:5’-AAGAGGGCTGGAAGAAGGAG-3’	
	R:5’-TCTCCTTGACATTGGGGTTC-3’	

Table S10.

Primer sequences of genes mentioned in the article of qPCR (Rat)

Target genes	Primer sequences(5’ → 3’)	Amplification condition
Actin	F:5’- GGCCAACCGTGAAAAGATGACC-3’<br>R:5’- AACCCTCATAGATGGGCACAG’	Stage 1: 95°C, 10s<br>Stage 2: 95°C, 10s<br>60°C, 20s<br>72°C, 20s<br>Number of cycles: 40<br>Stage 3: 95°C, 5s<br>65°C, 1min<br>4°C, ∞
Agt	F:5’-CGCCTAAAACAGCCATTTGT-3’<br>R:5’-ACCCCCTCTAGTGGCAAGTT-3’	
Serpine1	F:5’-TGGTGAACGCCCTCTATTTC-3’<br>R:5’-TAGGGCAGTTCCAGGATGTC-3’	
Lep	F:5’-GAGACCTCCTCCATCTGCTG-3’<br>R:5’-CATTCAGGGCTAAGGTCCAA-3’	
Il2	F:5’-CCCTGCAAAGGAAACACAGC-3’<br>R:5’-CGTGAGCATCATGGGGAGTT-3’	
Cyp19a1	F:5’-CAGAGTATCCGGAGGTGGAA-3’<br>R:5’-ACTCGAGCCTGTGCATTCTT-3’	
Srd5a1	F:5’-TGCTCGACATGCTGGTCTAC-3’<br>R:5’-GGCTGCAGGACGAATGTACT-3’	
Hsd3b	F:5’-GGTGCAGGAGAAAGAACTGC-3’<br>R:5’-CGGTGTGGATGACAACAGAG-3’	
Cyp11a1	F:5’-TCACATGCAGAATTTCCAGAAG-3’<br>R:5’-AGGATGTAACTGACTCCATGTTG-3’	

Star	F:5’-CACAGTCATCACCCATGAGC-3’
	R:5’-AGCTCTGATGACACCGCTTT-3’

Table S11.

siRNA sequences of genes mentioned in the article

Oligo	sequences（5’-3’）	
rat-SERPINE1-si-1	sense	GGUUCUGGUCUUUGGGAAAdTdT
	antisense	UUUCCCAAAGACCAGAACCdTdT
rat-SERPINE1-si-2	sense	CAGCAGAUCCAAGAUGCUAdTdT
	antisense	UAGCAUCUUGGAUCUGCUGdTdT
rat-SERPINE1-si-3	sense	GCUCAGAACAACAAGUUCAdTdT
	antisense	UGAACUUGUUGUUCUGAGCdTdT
hs-SERPINE1-si-1	sense	GAUUCAAGAUUGAUGACAAdTdT
	antisense	UUGUCAUCAAUCUUGAAUCdTdT
hs-SERPINE1-si-2	sense	AGACCAACAAGUUCAACUAdTdT
	antisense	UAGUUGAACUUGUUGGUCUdTdT
hs-SERPINE1-si-3	sense	CGACAUGUUCAGACAGUUUdTdT
	antisense	AAACUGUCUGAACAUGUCGdTdT

Table S12.

List of antibodies

Peptide/ protein target	Host	Dilution used	Manufacturer	Catalog
Beta Actin Antibody	Mouse Monoclonal	1:3000	Proteintech Group Inc	Cat #: 66009-1-lg; RRID: AB_2782959
DNMT1 antibody	Rabbit Polyclonal	1:1000 for WB	Abcam	Cat #: ab19905; RRID: AB_731983
DNMT3A (E9P2F) Rabbit mAb	Rabbit Monoclonal	1:50 for ChIP	Cell Signaling Technology	Cat #: 49768; RRID: AB_2799365
CYP19 (E-9)	Mouse Monoclonal	1:500 for KGN cell	Santa Cruz Biotechnology	Cat #: sc-374176; RRID: AB_10986411
Anti-Aromatase antibody - C-terminal	Rabbit Polyclonal	1:1000 for rat GCs	Abcam	Cat #: ab191093; RRID: AB_2737021
PAI-1 antibody	Rabbit Polyclonal	1:1000 for KGN cell	Proteintech Group Inc	Cat #: 13801-1-AP; RRID: AB_2186881
Anti-PAI1 antibody [EPR21850-82]	Rabbit Monoclonal	1:1000 for rat GCs	Abcam	Cat #: ab222754; RRID: N/A

SRD5A1 antibody	Rabbit Polyclonal	1:1000	Proteintech Group Inc	Cat #: 26001-1-AP; RRID: AB_2880328
Androgen Receptor (D6F11) XP Rabbit mAb	Rabbit Monoclonal	1:1000	Cell Signaling Technology	Cat #: 5153; RRID: AB_10691711
LHCGR antibody	Rabbit Polyclonal	1:1000	Proteintech Group Inc	Cat #: 19968-1-AP; RRID: AB_10697685
FSHR antibody	Rabbit Polyclonal	1:1000	Proteintech Group Inc	Cat #: 22665-1-AP; RRID: AB_2631204
Akt (pan) (40D4) Mouse mAb	Mouse Monoclonal	1:1000	Cell Signaling Technology	Cat #: 2920; RRID: AB_1147620
Phospho-Akt (Ser473) (D9E) XP® Rabbit mAb	Rabbit Monoclonal	1:1000	Cell Signaling Technology	Cat #: 4060; RRID: AB_2315049
CREB (48H2) Rabbit mAb	Rabbit Monoclonal	1:1000	Cell Signaling Technology	Cat #: 9197; RRID: AB_331277
Phospho-CREB (Ser133)	Rabbit Monoclonal	1:1000	Cell Signaling Technology	Cat #: 9198; RRID: AB_2561044

(87G3) Rabbit mAb				
FoxO1 (D7C1H)	Mouse Monoclonal	1:1000	Cell Signaling Technology	Cat #: 14952; RRID: AB_2722487
Phospho-FoxO1 (Ser256) Antibody	Rabbit Monoclonal	1:1000	Cell Signaling Technology	Cat #: 9461; RRID: AB_329831
PKA C-α Antibody	Rabbit	1:1000	Cell Signaling Technology	Cat #: 4782; RRID: AB_2170170
Histone H3 (D1H2) XP Rabbit mAb	Rabbit Monoclonal	1:1000	Cell Signaling Technology	Cat #: 4499; RRID: AB_10544537
PCNA (D3H8P) XP® Rabbit mAb	Mouse Monoclonal	1:1000	Cell Signaling Technology	Cat #: 13110; RRID: AB_2636979
